# Prevalence and associated risk factors of intestinal parasites among schoolchildren in Ecuador, with emphasis on the molecular diversity of *Giardia duodenalis*, *Blastocystis* sp. and *Enterocytozoon bieneusi*

**DOI:** 10.1371/journal.pntd.0011339

**Published:** 2023-05-24

**Authors:** Estephany Tapia-Veloz, Mónica Gozalbo, Marisa Guillén, Alejandro Dashti, Begoña Bailo, Pamela C. Köster, Mónica Santín, David Carmena, María Trelis

**Affiliations:** 1 Area of Parasitology, Department of Pharmacy and Pharmaceutical Technology and Parasitology, University of Valencia, Valencia, Spain; 2 Department of Medicine and Public Health, Science of the Food, Toxicology and Legal Medicine, University of Valencia, Valencia, Spain; 3 Parasitology Reference and Research Laboratory, Spanish National Centre for Microbiology, Health Institute Carlos III, Majadahonda, Spain; 4 Environmental Microbial and Food Safety Laboratory, Agricultural Research Service, United States Department of Agriculture, Beltsville, Maryland, United States of America; 5 Center for Biomedical Research Network (CIBER) in Infectious Diseases, Health Institute Carlos III, Madrid, Spain; 6 Joint Research Unit on Endocrinology, Nutrition and Clinical Dietetics, University of Valencia-Health Research Institute La Fe, Valencia, Spain; Wadsworth Center, UNITED STATES

## Abstract

**Background:**

Intestinal helminths, including Soil-Transmitted Helminth (STH), and Gastrointestinal Protist (GP) infections are major contributors to the global burden of disease, particularly in low-income countries such Ecuador. Their epidemiology in these settings is largely unknown.

**Methodology:**

This prospective cross-sectional study investigates the carriage of intestinal helminths, including STH, and GP in asymptomatic schoolchildren (3–11 years) in the Chimborazo and Guayas provinces, Ecuador. Single stool samples (*n* = 372) and epidemiological questionnaires on demographics and potential risk factors were collected from participating schoolchildren. Conventional microscopy examination was used as screening method, and molecular (PCR and Sanger sequencing) assays were used to further investigate the epidemiology of some GP. A multivariate logistic regression analysis was used to evaluate the strength of the association of suspected risk factors with the presence of helminths and GP.

**Principal findings:**

At least one intestinal parasite species was observed by microscopy in 63.2% (235/372) of the participating schoolchildren. *Enterobius vermicularis* (16.7%, 62/372; 95% CI: 13.0–20.9) and *Blastocystis* sp. (39.2%, 146/372; 95% CI: 34.2–44.2) were the most prevalent among helminths and GP, respectively. Assemblages A (50.0%), B (37.5%) and A+B (12.5%) were detected within *Giardia duodenalis* and ST3 (28.6%), ST1 and ST2 (26.2% each), and ST4 (14.3%) within *Blastocystis* sp. Three genotypes, two known (A: 66.7%; KB-1: 16.7%) and a novel (HhEcEb1, 16.7%) were identified within *Enterocytozoon bieneusi*. Municipality of origin, household overcrowding, and poor sanitation and personal hygiene habits were risk factors for childhood intestinal parasites colonization.

**Conclusions/Significance:**

Despite massive government drug administration programs, STH and GP infection remain a public health concern in paediatric populations living in poor-resource settings. Molecular analytical methods are required to better understand the epidemiology of these intestinal parasites. This study provides novel information on the occurrence of *Blastocystis* sp. and *E*. *bieneusi* genetic variants circulating in Ecuadorian human populations.

## Author summary

Intestinal parasitic infections remain a public health concern in poor-resource areas in Ecuador, primarily affecting paediatric populations. Our results indicate that mass drug administration programs targeting intestinal helminths, including STH, are insufficient to effectively control the spreading and perpetuation of these pathogens in deprived communities. These interventions must be complemented with measures directed to improve household quality and living conditions, sanitation, and health education for schoolchildren and their families. Together with access to safe drinking water, these measures are necessary to reduce infections with STH and GP in poor communities in Ecuador. Under the One Health approach, more research should be conducted to ascertain to which extent the animal reservoirs (livestock, wildlife) and contaminated environmental (water, soil, grass) contribute to the burden of human disease caused by intestinal parasites.

## Introduction

Intestinal helminths, including Soil-Transmitted Helminth (STH), and Gastrointestinal Protist (GP) infections are major contributors to the global burden of disease, particularly in resource-deprived, low-income countries from tropical and subtropical regions [1–3]. In these endemic areas, concomitant infections are common, and their synergistic effect might exacerbate detrimental health outcomes in infected individuals [4]. Chronic or repeated STH and GP infections during childhood have a strong link with stunting, the most common form of malnutrition in children aged under five years [5]. In paediatric populations, stunting or chronic malnutrition refers to both reduced physical growth and cognitive impairment, representing a major public health concern leading to lifelong adverse health, education, and economic outcomes [6]. Because of their chronic rather than acute nature, STH infections are only partially addressed in the Global Burden of Disease (GBD) studies [7], whereas diarrhoea-causing GP infections are not formally considered as neglected tropical diseases [8]. These limitations impair our understanding of the epidemiology of STH and GP in endemic areas.

STH including hookworms (*Ancylostoma duodenale*, *Necator americanus*), roundworms (*Ascaris lumbricoides*, *Strongyloides stercoralis*), and whipworms (*Trichuris trichiura*) are parasitic nematodes that infect humans through contact with soil contaminated with parasite eggs or infective larval stages [9]. It is estimated that 820 million people are infected with *A*. *lumbricoides*, 460 million with *T*. *trichiura*, 460 million with *N*. *americanus* and *A*. *duodenale*, and 386 million with *S*. *stercoralis* in 102 countries worldwide [10, 11]. These infections contributed to an estimated 3.4-million Disability-Adjusted Life-Years (DALYs) and 6,000 deaths [12].

Among GP, the protozoa *Cryptosporidium* spp., *Giardia duodenalis*, and *Entamoeba histolytica* are regarded as relevant diarrhoea-causing pathogens globally. They are faecal-orally transmitted either directly through contact with infected humans and other animals, or indirectly via ingestion of contaminated food or water. Cryptosporidiosis is the second cause of childhood mortality after rotaviral enteritis in sub-Saharan African and southwest Asian countries [13] causing an estimated 48 million DALYs [14]. Although rarely mortal, giardiasis affects 200 million people globally every year, mainly children aged between two and five years [15]. *Entamoeba histolytica* has been linked to an increased risk of death in infants and toddlers with moderate-to-severe diarrhoea in sub-Saharan Africa [16]. Other less frequent GP, but still of public health relevance, include the microsporidia *Enterocytozoon bieneusi* and the Stramenopile *Blastocystis* sp. The former is an opportunistic pathogen primarily infecting immunocompromised individuals [17], whereas the latter has been increasingly linked to a variety of intestinal (diarrhoea, irritable bowel syndrome) and extra-intestinal (urticaria) clinical manifestations [18]. All the GPs mentioned above are featured by a large intra-species molecular diversity that influence their host range and specificity, pathogenicity, virulence, and zoonotic potential. Therefore, assessing the species/subtypes/genotypes involved is essential to characterise the epidemiology of these parasites [19–21].

As a shared feature, intestinal helminths and GP infections are highly endemic amongst people who are poor with little or no access to basic services and infrastructures. Under these circumstances, environmental improvements including access to safe drinking water, basic sanitation, and hygiene have been demonstrated effective to reduce the burden of those infections [12, 22–24].

In Ecuador, intestinal parasitic infections are widespread. *A*. *lumbricoides* (7–45%) and *T*. *trichiura* (3–25%) are the most common STH (S1 Table) [25–32]. Regarding GP, the most prevalent species are *G*. *duodenalis* (4–40%) and members of the *Entamoeba* complex (12–34%). *Cryptosporidium* spp. (3–14%) and *Blastocystis* sp. (7–81%) are less commonly documented, although sometimes at high prevalence rates (S2 Table) [25–40]. When considered together, these data indicate that 80% of the rural and 40% of the urban-marginal populations in Ecuador are affected by intestinal parasites. They rank second in the list of the main causes of ambulatory morbidity and within the top ten causes of paediatric [25, 28, 38]. Most of the helminths and GP colonization rates reported in these studies were found in apparently healthy children and using microscopy examination as screening test. Molecular genotyping data is available from few studies and only for *G*. *duodenalis* and *Blastocystis* sp. [34, 37, 39].

This aim of this study was to investigate the prevalence and associated risk factors of intestinal helminths and GP in schoolchildren from the Chimborazo and Guayas provinces, two regions were little or no information at all was available on the epidemiology of these pathogens. Microscopy was conducted for all samples collected and PCR-based methods for molecular characterization of some relevant GPs.

## Methods

### Ethics statement

This study was approved by the Ethics Committee for Human Research of the University of Valencia (procedure number: H1518738039128, 1 March 2018), by the Ethical Subcommittee for Research on Human Subjects of the Central University of Ecuador-SEISH-UCE (procedure number: 001-UV-2019, 22 January 2019), by the National Directorate of Prevention and Control Strategies and the National Directorate of Health Promotion of the Ministry of Public Health-MSP of Ecuador (procedure number: MSP-DIS-2020-0219-O, 18 May 2020) to meet the principles established by the Helsinki Declaration and by the Spanish/Ecuadorian legislations regarding biomedical research and personal data protection. Formal written consent was obtained from each parent/guardian of the participants.

### Study design and setting

A prospective cross-sectional study was conducted in asymptomatic schoolchildren (3–11 years) in the Ecuadorian municipalities of Penipe and Pallatanga (in the Sierra region of the Chimborazo province) and General Antonio Elizalde (GAE, in the Coast region of the Guayas province). Stool samples were collected from participating schoolchildren from June to August 2020 and analysed for the presence of intestinal parasites by microscopy. In parallel, molecular (PCR and Sanger sequencing) methods were used to detect and genotype the main diarrhoea-causing protists species (*Entamoeba* spp., *G*. *duodenalis*, *E*. *bieneusi*, *Blastocystis* sp., and *Cryptosporidium* spp.).

**Fig 1:**
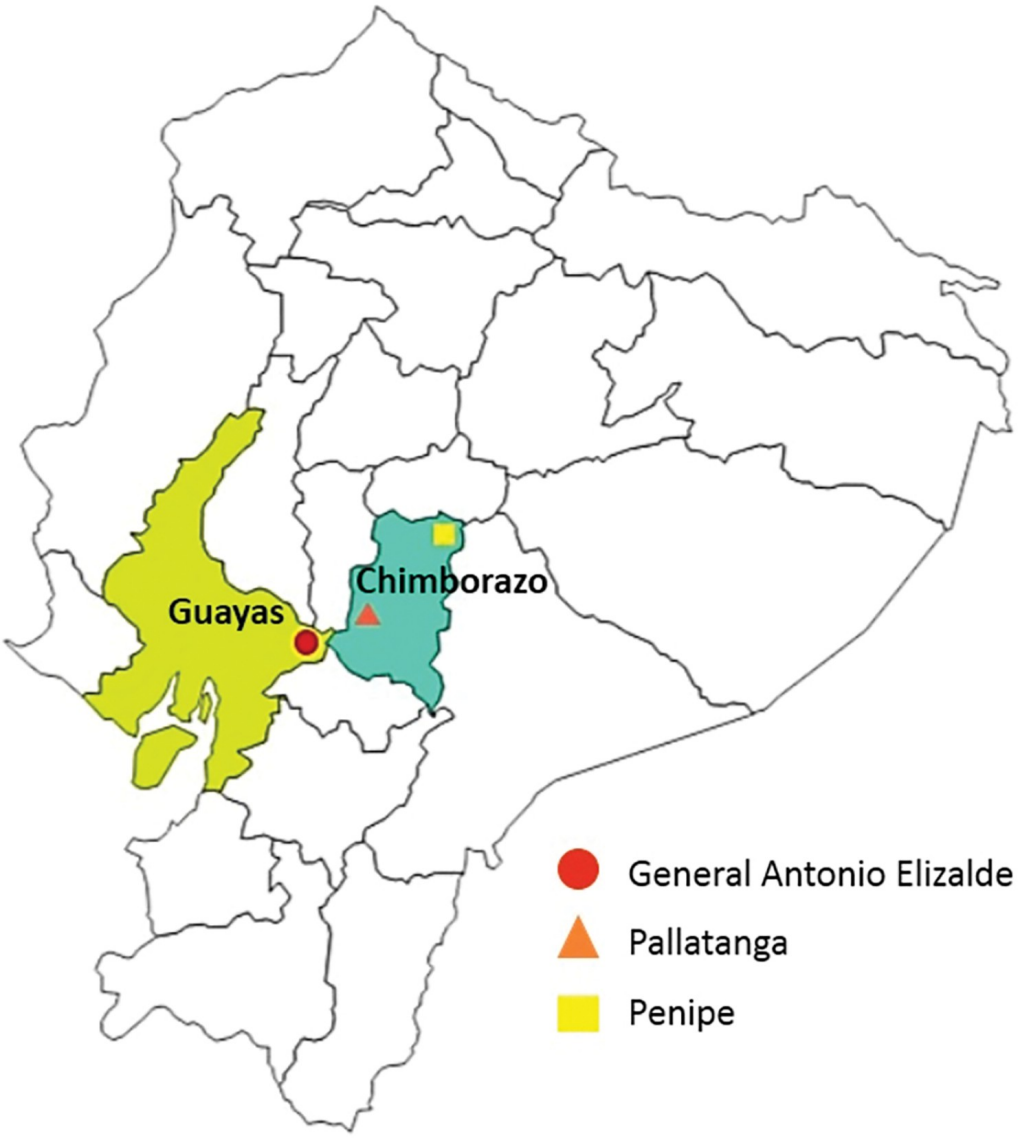
Map of Ecuador. The geographical localization of the three municipalities surveyed in the present study in Chimborazo and Guayas provinces are indicated.

The three surveyed municipalities differ from each other in geographic, climatic, demographic, and socioeconomic factors, providing a representative picture of the life conditions in the country (Fig 1). Penipe [meters above sea level (MASL): 2,500–5,424] has a cold [average annual temperature (AAT): 14°C], mountainous climate; Pallatanga (MASL: 1,200–1,462) has a temperate sub-humid mountainous climate (AAT: 20°C) and GAE (MASL: 300–700) has a humid tropical climate (AAT: 24°C).

In order to contextualise the reality of each municipality analysed and according to the available data, it should be noted that Pallatanga has a Gini coefficient, which is a measure to represent income inequality within a social group, of 0.37; Penipe of 0.35; and GAE of 0.30 [41]. The mean number of years of schooling of the population in Pallatanga is 6.2 years; in Penipe, it is 7.7 years; and in GAE, it is 8.3 years [41, 42]. The percentage of households with inadequate sanitation facilities in Pallatanga is 14.9%; in Penipe 11.4%, and in GAE 5.7%. The percentage of homes with a public water supply in Pallatanga is of 44.8%, in Penipe of 76.6%, and in GAE of 63.2% [43].

### Sample collection

Schools were approached in collaboration with the local university (Escuela Superior Politécnica de Chimborazo, ESPOCH). School heads were contacted and invited to participate in the study. Three public primary schools agreed to participate each with 355–510 schoolchildren. There were 372 schoolchildren enrolled, aged 3–11 years (median: 8.0 years), of which 115 were enrolled in GAE, 161 in Pallatanga, and 96 in Penipe. The rate ratio of boys/girls was 0.86. All recruited schoolchildren were from communities of low-to-medium socioeconomic status.

Informative meetings were personally held with teachers, who explained the goals and procedures of the project to schoolchildren. Parents/legal guardians were informed by formal letter. Participating schoolchildren were provided with uniquely labelled sampling kits (10 mL polystyrene plastic tube, disposable spatula, Graham tape, and instructions) to obtain individual samples. Parents/legal guardians assisted in the collection of samples from consenting schoolchildren and brought the samples to school the next day. A group of 10 students of the ESPOCH were trained to assist in the sample collection. Collected samples were immediately transported at the Chemistry Laboratory of ESPOCH and stored at 4°C until further processing.

### Epidemiological questionnaire

A standardized questionnaire (S3 Table) was provided as part of the sampling kit to be completed by the children’s parents/legal guardians. Questions included: (i) demographic characteristics (age, gender, municipality of residence, number of siblings, number of relatives residing at home); (ii) behavioural habits (consumption of unsafe water, hand and fruit/vegetable washing, contact with domestic animals, playing outdoors); and (iii) clinical manifestations (diarrhoea, constipation, abdominal pain, vomiting, weight loss, allergic manifestations, teeth grinding, anal itching, abdominal distention, and flatulence). Completed questionnaires and written informed consents signed by the parent/guardian were returned for collection by each participating schoolchild as described above.

### Parasitological assessment

An aliquot of fresh stool samples from each participant was analysed at the Chemistry Laboratory of ESPOCH using the Kato-Katz technique for the detection, identification, and quantitation of intestinal helminths. The remaining faecal material was preserved in 70% ethanol and, together with the Graham tapes, shipped to the Parasitology Laboratory of the Faculty of Pharmacy, University of Valencia (Spain) for further analyses.

A 3-gram stool sample from each participant was concentrated and filtered for 5 min at 2,500 rpm using Midi Parasep filter devices (Apacor Ltd., Wokingham, UK). The sediment obtained was divided in two aliquots. One was used for DNA extraction (see below); the other was fixed with 10% formalin in a 1:3 proportion for microscopic examination; and with a small amount of the remaining sediment thin smears were also prepared and stained with the Modified Ziehl-Neelsen (MZN) for the detection and identification of *Cryptosporidium* spp. and other coccidian. Graham tapes were examined by 10x microscopy for detecting eggs of *Enterobius vermicularis*. A sample was considered negative when no parasite structures (eggs, larvae cysts, oocysts or trophozoites) were observed.

### DNA extraction and purification

Genomic DNA was isolated from 200 mg of each stool sample using the QIAamp DNA Stool Mini Kit (Qiagen, Hilden, Germany) according to the manufacturer’s instructions. Extracted and purified DNA was eluted in 100 μL of PCR-grade water and kept at 4°C until further molecular analysis.

### Molecular detection and characterization of intestinal protists

Molecular studies focused on the most prominent GP species studied as a cause of diarrhoea and those under discussion or emergency. The presence of *G*. *duodenalis* was investigated using a real-time PCR (qPCR) method targeting a 62-bp region of the small subunit of the rRNA (*ssu* rRNA) gene of the parasite as the initial screening method [44]. For assessing the molecular diversity of this protozoa at the assemblage and sub-assemblage levels, a sequence-based multilocus genotyping (MLST) scheme targeting the genes encoding for the glutamate dehydrogenase (*gdh*), β-giardin (*bg*), and triose phosphate isomerase (*tpi*) proteins was adopted. Only samples that yielded cycle threshold (C_T_) values <32 in qPCR were assessed under this MLST scheme. A semi-nested PCR was used to amplify a 432-bp fragment of the *gdh* gene [45], and nested PCRs were used to amplify 511 and 530 bp fragments of the *bg* and *tpi* genes, respectively [46, 47]. Detection and differential diagnosis between pathogenic *E*. *histolytica* and non-pathogenic *E*. *dispar* was carried out by a qPCR method targeting a 172-bp fragment of the *ssu* rRNA gene of the *E*. *histolytica*/*E*. *dispar* complex [48, 49]. The presence of *Cryptosporidium* spp. was assessed using a nested-PCR protocol to amplify a 587 bp fragment of the *ssu* rRNA gene of the parasite [50]. Identification of *Blastocystis* sp. was achieved by a direct PCR protocol targeting a 600 bp fragment of the *ssu* rRNA gene of the protist [51]. Detection of *E*. *bieneusi* was conducted by a nested PCR protocol to amplify the internal transcribed spacer (ITS) region as well as portions of the flanking large and small subunit of the ribosomal RNA gene as previously described [52]. Detailed information on the oligonucleotides and the PCR conditions used for the molecular identification and/or characterisation of the unicellular parasites investigated in the present study is presented in S4 and S5 Tables, respectively.

All the qPCR protocols described above were conducted on a Corbett Rotor Gene 6000 real-time PCR system (Qiagen). Reaction mixes always included 2x TaqMan Gene Expression Master Mix (Applied Biosystems, CA, USA). All the direct, semi-nested, and nested PCR protocols described above were conducted on a 2720 Thermal Cycler (Applied Biosystems). Reaction mixes always included 2.5 units of MyTAQ DNA polymerase (Bioline GmbH, Luckenwalde, Germany), and 5x MyTAQ Reaction Buffer containing 5 mM dNTPs and 15 mM MgCl_2_. Laboratory-confirmed positive and negative DNA samples of human origin for each parasitic species investigated were routinely used as controls and included in each round of PCR. PCR amplicons were visualized on 1.5–2% agarose gels (Conda, Madrid, Spain) stained with Pronasafe (Conda) nucleic acid staining solution. A 100 bp DNA ladder (Boehringer Mannheim GmbH, Baden-Wurttemberg, Germany) was used for the sizing of obtained amplicons.

### Sequence analyses

PCR products of the expected size on agarose gel were directly sequenced in both directions using appropriate internal primer sets (S4 Table). DNA sequencing was conducted by capillary electrophoresis using the BigDye Terminator chemistry (Applied Biosystems) on an on ABI PRISM 3130 automated DNA sequencer.

Raw sequencing data in both forward and reverse directions were viewed using the Chromas Lite version 2.1 (Technelysium Pty Ltd., South Brisbane, Australia) sequence analysis program (https://technelysium.com.au/wp/chromas/). The BLAST tool (http://blast.ncbi.nlm.nih.gov/Blast.cgi) was used to compare nucleotide sequences with sequences retrieved from the NCBI GenBank database. Generated DNA consensus sequences were aligned to appropriate reference sequences using the MEGA version 10 software [53] to identify *Giardia* species and assemblages/sub-assemblages, *Cryptosporidium* species and *E*. *bieneusi* genotypes.

*Blastocystis* sequences were submitted at the *Blastocystis* 18S database (https://pubmlst.org/organisms/blastocystis-spp) for subtype confirmation and allele identification. The sequences obtained in this study have been deposited in GenBank under accession numbers ON866725-ON866736 (*G*. *duodenalis*), ON858734-ON858742 (*Blastocystis* sp.), and OQ267689-OQ267691 (*E*. *bieneusi*).

### Phylogenetic analysis

Nucleotide sequences obtained in this study and *E*. *bieneusi* nucleotide sequences for genotypes previously identified in humans and animals as well as appropriate reference sequences to include all *E*. *bieneusi* groups retrieved from GenBank were aligned with the Clustal W algorithm using MEGA X [53]. Phylogenetic inference was carried out by the Neighbor-Joining (NJ) method as previously described [54]. Genetic distance was calculated with the Kimura parameter-2 model using MEGA X [53].

### Data analysis

Descriptive statistics were calculated, including measures of central tendency (mean and median), measures of dispersion (standard deviation, range, and coefficient of variation) and measures of shape (asymmetry and pointing) for quantitative variables, as well as the absolute and relative frequencies for the qualitative variables. Association analyses were performed, stratifying by gender and age to observe possible heterogeneity in the results according to these factors. When data were stratified and the sample size was small (*n* < 15), non-parametric tests were used (Fisher’s exact test and Mann–Whitney U test). To test the null hypotheses of no association between intestinal parasitism and demographic and socioeconomic factors and lifestyle habits controlling by confounders or effect modifiers, multiple logistic regression analyses with dummy variables for categorical terms, were applied. The magnitude of the association was expressed as adjusted odds ratios (AORs) with a 95% confidence interval (CI). A *P*-value ≤0.05 was considered statistically significant. All the variables were analysed using SPSS software version 26.0 (Statistical Package for Social Sciences, Chicago, IL, USA).

## Results

### Occurrence of intestinal parasites by optical microscopy

Overall, 63.2% (235/372) of the participating schoolchildren were infected/colonized by at least one intestinal parasite. Global prevalence rates for each sampling site varied from 41.7% (48/115) in GAE, 71.9% (69/96) in Penipe and 73.3% (118/161) in Pallatanga. The frequency of intestinal helminths and protists by optical microscopy in the surveyed schoolchildren by municipalities is shown in Table 1.

**Table 1:** Frequencies (%) of infection/colonization by helminthic and protist species detected by microscopy according to the municipality of origin. P-values in bold indicate statistical significance (P≤0.05).

Species	GAE (*n* = 115)	Penipe (*n* = 96)	Pallatanga (*n* = 161)	Total (*n* = 372)	*P*-value
*E*. *vermicularis*	9.6	9.4	26.1	16.7	**0.001**
*A*. *lumbricoides*	4.3	4.2	16.1	9.4	**0.001**
*T*. *trichiura*	1.7	2.1	9.3	5.1	**0.006**
*H*. *nana*	0.0	0.0	1.9	0.8	0.136
*Blastocystis* sp.	28.7	47.9	41.6	39.2	**0.012**
*E*. *coli*	7.0	39.6	32.3	26.3	0.000
*G*. *duodenalis*	7.0	18.8	13.0	12.6	**0.036**
*Entamoeba* complex^a^	0.9	4.2	10.6	5.9	**0.002**
*E*. *nana*	1.7	8.3	5.0	4.8	0.084
*E*. *hartmanni*	1.7	4.2	6.2	4.3	0.195
*C*. *mesnili*	0.0	2.1	0.0	0.5	0.056
*D*. *fragilis*	0.0	1.0	0.0	0.3	0.237
*Cryptosporidium* spp.	0.0	0.0	0.0	0.0	–

^a^*Entamoeba* complex: *E*. *histolytica*/*E*. *dispar*/*E*. *moshkovskii*/*E*. *bangladeshi*.

The most prevalent helminths were nematodes such as the human pinworm, *E*. *vermicularis* (16.7%, 62/372; 95% CI: 13.0–20.9), followed by the STHs, *A*. *lumbricoides* (9.4%, 35/372; 95% CI: 6.6–12.8) and *T*. *trichiura* (5.1%, 19/372; 95% CI: 3.1–7.9). *Hymenolepis nana*, the only cestode reported in the study, was detected at very low (<1%) infection rates and only in one of the schools in Pallatanga. Among GP, *Blastocystis* sp. was the most common protist identified (39.2%, 146/372; 95% CI: 34.2–44.2), followed by *Entamoeba coli* (26.3%, 98/372; 95% CI: 21.9–31.1), *G*. *duodenalis* (12.6%, 47/372; 95% CI: 9.4–16.4), and the *Entamoeba* complex (5.9%, 22/372; 95% CI: 3.7–8.8). Other species were identified at low (<5%, *Endolimax nana* and *Entamoeba hartmanni*) or very low (<1%, *Chilomastix mesnili* and *Dientamoeba fragilis*) infection/carriage rates. None of the 372 samples tested positive for *Cryptosporidium* spp. Prevalence rates among sampling sites ranged between 9.4–26.1% for *E*. *vermicularis*, 4.2–16.1% for *A*. *lumbricoides*, 1.7–9.3% for *T*. *trichiura*, 28.7–47.9% for *Blastocystis* sp., 7.0–39.6% for *Entamoeba coli*, 7.0–18.8% for *G*. *duodenalis*, and 0.9–10.6% for the members of the *Entamoeba* complex (Table 1).

Of 372 samples, a single parasite species was detected in 113 (30.4%). Multiparasitism with two species were found in 43 samples (11.6%) of which, 14 (32.6%) combined *Blastocystis* sp. + *E*. *coli*, 4 (9.3%) *Blastocystis* sp. + *E*. *vermicularis*, and 4 (9.3%) *Blastocystis* sp. + *G*. *duodenalis*. Seventy-seven samples (20.7%) presented multiparasitism with more than three species per individual, being the most common combination *Blastocystis* sp. + *G*. *duodenalis* + *E*. *coli* (9.3%).

Schoolchildren in Pallatanga were more likely to harbour helminths including *E*. *vermicularis*, *H*. *nana*, *A*. *lumbricoides* and *T*. *trichiura*. Schoolchildren in GAE were significantly less infected by GP including *Blastocystis* sp., *E*. *coli*, *G*. *duodenalis* and members of the *Entamoeba* complex than their counterparts in Pallatanga and Penipe (Table 1).

### Occurrence of intestinal protists by molecular analysis techniques

In addition to microscopy, faecal DNAs from all 372 stool samples collected were investigated by PCR for the presence of protists *Cryptosporidium* spp., *E*. *histolytica*, *G*. *duodenalis*; Stramenopile *Blastocystis* sp., and Microsporidia *E*. *bieneusi*. The frequency of the unicellular parasites analysed by molecular techniques in the surveyed population is shown in Table 2. With the new molecular results, and for the selected species, 50.5% (188/372) of participants were harbouring at least one GP. In this case, global prevalence rates for each sampling site varied from 43.5% (50/115) in GAE, 50.9% (82/161) in Pallatanga, and 58.3% (56/96) in Penipe.

**Table 2:** Frequencies (%) of infection/colonization by protist species investigated by PCR in the studied population according to the municipality of origin.

Species	GAE (*n* = 115)	Penipe (*n* = 96)	Pallatanga (*n* = 161)	Total (*n* = 372)	*P*-value
*G*. *duodenalis*	31.3	31.3	20.5	26.6	0.066
*Blastocystis* sp.	11.3	37.5	21.7	22.6	0.000
*E*. *dispar*	4.3	9.4	24.8	14.5	0.000
*E*. *bieneusi*	1.7	0.0	2.5	1.6	0.308
*Cryptosporidium* spp.	0.0	0.0	0.0	0.0	–
*E*. *histolytica*	0.0	0.0	0.0	0.0	–

PCR-based data, for the selected species, showed marked differences with microscopy-based data. Thus, *G*. *duodenalis* (26.6%, 99/372; 95% CI: 22.2–31.4) was the most prevalent species found, followed by *Blastocystis* sp. (22.6%, 84/372; 95% CI: 18.4–27.2), *E*. *dispar* (14.5%, 54/372; 95% CI: 11.1–18.5), and *E*. *bieneusi* (1.6%, 6/372; 95% CI: 0.6–3.5). Neither *Cryptosporidium* spp. nor *E*. *histolytica* were detected in the surveyed population. Prevalence rates among sampling sites ranged between 20.5–31.3% for *G*. *duodenalis*, 11.3–37.5% for *Blastocystis* sp., 4.3–24.8% for *E*. *dispar*, and 0.0–2.5% for *E*. *bieneusi* (Table 2). Remarkably, *G*. *duodenalis* infection rates were two-fold higher by PCR than by conventional microscopy (26.6% *vs*.12.6%), but the opposite result was observed for *Blastocystis* sp. (22.6% *vs*. 39.2%).

### Molecular characterization of *Giardia duodenalis*

A total of 99 samples tested positive for *G*. *duodenalis* by qPCR. Generated C_T_ values had a median value of 35.3 (range: 19.0–44.0). Genotyping analyses were attempted in the 17.2% (17/99) of samples that yielded qPCR C_T_ values <32. Out of the 17 DNA isolates investigated, 35.3% (6/17), 23.5% (4/17), and 41.2% (7/17) yielded amplicons of the expected sizes at the *gdh*, *bg*, and *tpi* loci, respectively. Of them, 58.8% (10/17) were amplified at least at one single locus, whereas multi-locus genotyping data at the three loci were available for 17.6% (3/17) (Table 3).

**Table 3:** Multilocus genotyping results of the eight *G*. *duodenalis*-positive samples successfully genotyped.

Patient ID	*gdh*	*tpi*	*bg*	Assigned assemblage	Assigned sub-assemblage
EC1048	–	AII	–	A	AII
EC1092	–	AII	B	A+B	AII+B
EC1116	BIII	B	B	B	BIII
EC1117	BIII	BIII	B	B	BIII
EC1488	BIV	BIII	–	B	BIII+BIV
EC1559	AII	AII	–	A	AII
EC1653	AII	AII	AII	A	AII
EC1668	AI	–	–	A	AI

*gdh*: Glutamate dehydrogenase; *tpi*: Triose phosphate isomerase; *bg*: β-giardin.

Assemblage/sub-assemblage assignment was conducted by direct comparison of the sequencing results obtained at the three loci (*gdh*, *bg*, and *tpi*) investigated. Overall, assemblage A (50.0%, 4/8) was more prevalent than assemblage B (37.5%, 3/8). A mixed A+B infections was detected in one sample (12.5%, 1/8) (Table 3).

Out of the six *gdh* sequences, one (16.7%) and two (33.3%) were assigned to the sub-assemblages AI and AII, respectively, showing 100% identity with reference sequences L40509 and L40510. Assemblages BIII and BIV were identified in two (33.3%) and one (16.7%) isolates, respectively. The two BIII sequences differed by 3–7 SNPs with reference sequence AF069059. The only BIV sequence identified varied by a single SNP from reference sequence L40508 (Table 4).

**Table 4:** Diversity, frequency, and main molecular features of *Giardia duodenalis* sequences generated.

Assemblage	Sub-assemblage	No. isolates	Locus	Reference sequence	Stretch	SNPs	GenBank ID
A	AI	1	*gdh*	L40509	88─416	None	ON866725
	AII	2	*gdh*	L40510	64─491	None	ON866726
B	BIII	1	*gdh*	AF069059	77─460	T147Y, G150A, C309Y, C336Y, G372R, C375Y, T456C	ON866727
		1	*gdh*	AF069059	40─460	C309Y, G372R, T456C	ON866728
	BIV	1	*gdh*	L40508	76─479	C255T	ON866729
A	AII	1	*bg*	AY072723	1─594	None	ON866730
B	B	2	*bg*	AY072727	102─550	C165T, C309T, G421A	ON866731
		1	*bg*	AY072727	102─590	C165Y, C309Y	ON866732
A	AII	4	*tpi*	U57897	294─805	None	ON866733
B	BIII	1	*tpi*	AF069561	1─456	G48A, G105A, G267A	ON866734
		1	*tpi*	AF069561	1─456	G48R, G105R, C186Y, G267R, C447T	ON866735
		1	*tpi*	AF069561	1─456	G399A	ON866736

*bg*: beta-giardin; *gdh*: glutamate dehydrogenase; R: A/G; *tpi*: triose phosphate isomerase; Y: C/T. SNP: Single nucleotide polymorphisms.

Out of the four *bg* sequences, one (25.0%) was identified as sub-assemblage AII and was identical to reference sequence AY072723. The remaining three sequences (75.0%) were identified as assemblage B and differed from 2–3 SNPs from reference sequence AY072727. Because *bg* is unsuitable for intra-assemblage discrimination for assemblage B, no information at the sub-assemblage level was available at this locus (Table 4).

Finally, out of the seven *tpi* sequences, four (57.1%) were identified as sub-assemblage AII and were identical to reference sequence U57897. The remaining three sequences (42.9%) differed by 1–5 SNPs with reference sequence AF069561 (Table 4).

### Molecular characterization of *Blastocystis* sp

A total of 92 faecal DNA samples yielded amplicons of the expected size on agarose gels compatible with *Blastocystis* sp. Of them, 91.3% (84/92) were successfully subtyped. The remaining eight isolates produced unreadable or poor-quality Sanger sequences (associated to faint bands on agarose gels) and were conservatively not considered as true *Blastocystis*-positive samples.

The frequency and molecular diversity of *Blastocystis* sp. isolates at the *ssu* rRNA locus is described in Table 5. Four distinct *Blastocystis* subtypes (ST) were identified, being the most prevalent ST3 (28.6%, 24/84), followed by ST1 and ST2 (26.2%, 22/84 each), and ST4 (14.3%, 12/84). Mixed infections samples involving different STs of the protists were also identified in four samples (4.8%, 4/84), although the exact identity of the STs involved could not be determined due to the complexity of sequence trace chromatograms with mixed patterns.

**Table 5:** Diversity, frequency, and main molecular features of *Blastocystis* sp. *ssu* rRNA sequences (*ca*. 600 bp) generated in the present study.

Subtype	Allele	No. isolates	Reference sequence	GenBank ID
ST1	4	22	MN836826	ON858734
ST2	9	2	OL623671	ON858735
ST2	12	11	OK285237	ON858736
ST2	11+12	8	MF669067	ON858737
ST2	12+68	1	OK285237	ON858738
ST3	34	20	MT160370	ON858739
ST3	34+36	3	MN836837	ON858740
ST3	57	1	ON796017	ON858741
ST4	42	12	OK285228	ON858742

Regarding intra-subtype diversity, no diversity was observed for ST1 and ST4, all isolates belonged to alleles 4 and 42, respectively. In contrast, isolates assigned to ST2 and ST3 displayed a much larger genetic diversity. Thus, allele 12 was the most frequent genetic variant (50.0%, 11/22) found within ST2, followed by mixed infections involving alleles 11+12 (36.4%, 8/22), allele 9 (9.1%, 2/22) and a rare mixed infection by alleles 12+68 (4.5%, 1/22). Similarly, allele 34 accounted for most (83.3%, 20/24) of the genetic variants found within ST3, followed by mixed infections by alleles 34+36 (12.5%, 3/24), and allele 57 (4.2%, 1/24).

### Molecular characterization of *Enterocytozoon bieneusi*

**Fig 2:**
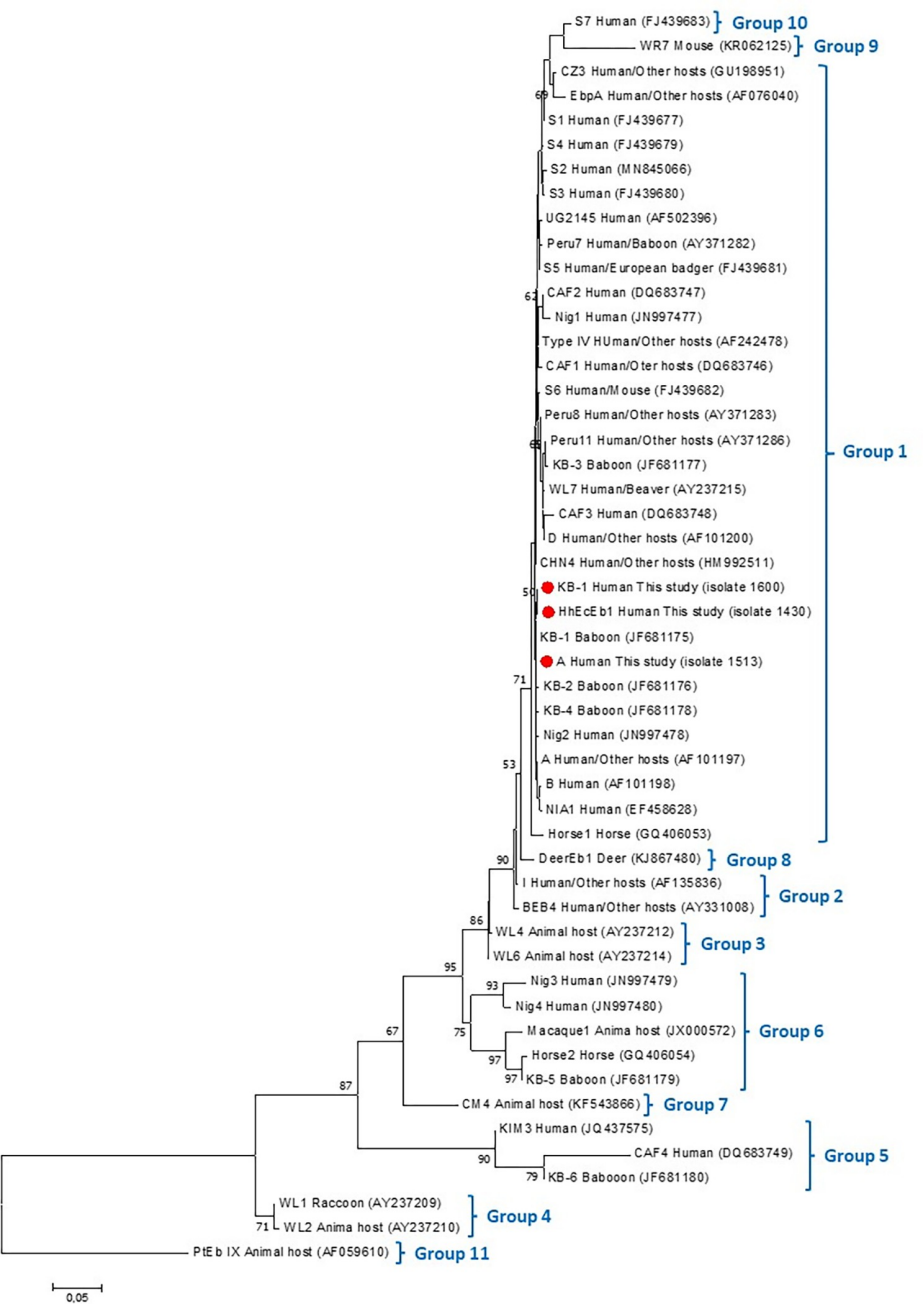
Phylogenetic relationships among *Enterocytozoon bieneusi* genotypes identified in this study. All genotypes identified in humans and animals, and genotypes to cover all groups of *E*. *bieneusi* were included for comparative purposes. Analyses were inferred by a Neighbor-Joining method of the entire ITS of rRNA gene based on genetic distances calculated by the Kimura two-parameter model (MEGA X software). All nucleotide sequences include host information with the GenBank accession number in parenthesis. Nucleotide sequences determined in this study are identified with red circles before the genotype name.

In the case of microsporidia, detection was performed by nested-PCR and only for *Enterocytozoon bieneusi* that is the most prevalent species causing human microsporidiosis [17]. Out of the six *Enterocytozoon*-positive isolates, four (66.7%, 4/6) were identified as genotype A, and one (16.7%, 1/6) as genotype KB-1, both showing 100% identity with reference sequences AF101197 and MN136778, respectively. The remaining isolate (16.7%, 1/6) corresponded to a novel genotype named HhEcEb1. The identity of this novel *E*. *bieneusi* genotype were confirmed in two independent PCR reactions. Novel HhEcEb1 differed from known genotype A by a single SNP (T to C) in position 158 of reference sequence AF101197. Phylogenetic analysis revealed that novel HhEcEb1clustered within zoonotic Group 1 (Fig 2).

### Risk factors of intestinal parasites

Logistic regression analyses were conducted to assess the risk of intestinal parasitism based on the demographic, socioeconomic, and behavioural features of the surveyed schoolchildren population. Multivariate logistic regression analysis computing the whole study population showed that residing in a particular municipality was the only variable determining the presence of intestinal parasites (S6 Table). Schoolchildren living in Pallatanga or Penipe were 2.2-fold more likely to harbour helminths and/or GP infections than those living in GAE (AOR = 2.296; 95% CI: 1.226–4.301, *P* = 0.009; AOR = 2.231; 95% CI: 1.067–4.665, *P* = 0.033).

When data were stratified by municipality of origin, other variables reached statistical significance and they could be identified as risk factors for intestinal parasites, including more than five persons residing in the same house in GAE (AOR = 22.634; 95% CI: 1.778–288.093, *P* = 0.016); and no handwashing after bathing (AOR = 0.54, 95% CI = 0.005–0.541, *P* = 0.013) and consumption of unsafe water (AOR = 0.127, 95% CI: 0.220–0.743, *P* = 0.022) in Penipe (S6 Table). None of the variables considered in the study increased the likelihood of being infected by intestinal parasites in Pallatanga.

Despite the fact that the children are school-going and apparently asymptomatic, the most prevalent clinical manifestations reported by the participating children were abdominal pain (40.6%, 151/372), flatulence (29.0%, 108/372), and diarrhoea (26.1%, 97/372).

## Discussion

We studied the epidemiology and risk factors for helminths and GP infections in schoolchildren from the Chimborazo and Guayas provinces, Ecuador. Although microscopy was used as screening test, molecular (PCR and Sanger sequencing) methods were also used for in-depth investigation of the main parasitic intestinal protist species. Six out of 10 children had at least one intestinal parasite species. *E*. *vermicularis*, followed by *A*. *lumbricoides* and *T*. *trichiura* were the most common species among helminths, and *Blastocystis* sp., *E*. *coli* and *G*. *duodenalis* (12.6%) among GP. Regarding molecular diversity, assemblages A and B were detected within *G*. *duodenalis*, and subtypes ST1-ST4 within *Blastocystis* sp. Three genotypes, two known (A, KB-1) and a novel (HhEcEb1) were identified within *E*. *bieneusi*. Several household and behavioural factors indicative of greater poverty and social marginalisation were associated with helminths and GP infection risk. Household overcrowding, consumption of unsafe water, and poor personal hygiene habits were risk factors for childhood intestinal parasitic diseases.

Strengths of this survey include a large sample size representative of three epidemiological scenarios with distinct environmental, climatic and socioeconomic conditions. The use of PCR-based methods for GP detection and genotyping allowed for i) improved prevalence estimates compared to more traditional (microscopy) methods, and ii) assessing potential sources of infection and transmission pathways. This is one of the main contributions of the study, as GP molecular data is particularly scarce in Ecuador. This study provides also the first description of microsporidiosis by *E*. *bieneusi* in the country.

The roundworm *A*. *lumbricoides* and the whipworm *T*. *trichiura* have been consistently reported as the most dominant STH present in Ecuadorian human populations, with infection varying from 7–45% (*A*. *lumbricoides*) and 0.5–25% (*T*. *trichiura*) (S1 Table). In contrast, we identified the presence of *A*. *lumbricoides* and *T*. *trichiura* at comparatively lower (9.4% and 5.1%, respectively) infection rates. This result is probably associated to Mass Drug Administration (MDA) campaigns with albendazol by the Ministry of Health. Remarkably, the pinworm *E*. *vermicularis* was the most prevalent nematode detected by the Graham test in the surveyed paediatric population (16.7%), a much higher rate than that (0.5%) identified in indigenous communities in Santo Domingo de los Tsáchilas province by conventional coproparasitological procedures [31]. The Graham’s perianal swab method is the recommended procedure for the detection of *E*. *vermicularis* in young children [55], these data suggest that the true prevalence of this helminth is likely much higher than initially anticipated.

Recent studies investigating the epidemiology of STH in Ecuador have provided interesting data. A longitudinal birth cohort study (new-borns to 8 years of age) has shown that infections with *A*. *lumbricoides* and *T*. *trichiura* peak between 3 and 5 years [56]. Marked differences in STH prevalence rates have also been documented nationwide. Thus, comparatively fewer *A*. *lumbricoides* and *T*. *trichiura* infections occur in coastal and highland areas than in the Amazon region where greater poverty and inadequate sanitation likely favour transmission [57, 58]. Local spatial clustering of STH infection has been observed among indigenous Shuar inhabiting two regions of Amazonian Ecuador [59]. This seems to be also the case of our study, where schoolchildren in Pallatanga or Penipe were at higher risk of infection by STH and/or GP than their counterparts in GAE.

Several environmental, sociodemographic, and behavioural factors have been linked to an increased risk of STH infection. *T*. *trichiura* infection intensity were lower among Shuar indigenous people living in houses with wood floors than those with dirt floors [60]. Indeed, household dust samples have been shown to contain DNA for a variety of STH including *S*. *stercoralis* (52%), *A*. *lumbricoides* (39%), *Toxocara* spp. (42%), hookworm (18%) and *T*. *trichiura* (8%) [61]. In the present study, determinants of infection risk included household overcrowded conditions, consumption of unsafe water, and inappropriate personal hygiene practices, highlighting the strong link between poverty and STH infections.

Regarding GPs, microscopy examination of faecal specimens allowed the identification of a well-recognized pathogen (*G*. *duodenalis*), two species of uncertain pathogenicity (*Blastocystis* sp. and *D*. *fragilis*), and commensals of the genera *Entamoeba* (*E*. *coli*, *E*. *dispar*, *E*. *hartmanni*), *Endolimax nana* and *Chilomastix mesnili*. *G*. *duodenalis* is widely present in Latin American countries, where microscopy-based prevalence rates in humans have been typically reported in the range of 10–50%, mostly in paediatric populations [62].

In Ecuador, *G*. *duodenalis* has been identified at prevalence rates of 26–33% in children with clinical manifestation, of 16–40% in asymptomatic children, and of 4–20% in rural and urban community-based surveys (S2 Table). In the present study, overall *G*. *duodenalis* prevalence was of 12.6% and of 26.6% by microscopy examination and qPCR, respectively. This finding confirm i) the superior diagnostic performance of molecular methods over conventional assays, and ii) the suitability of qPCR for the detection of subclinical *Giardia* spp. infections in endemic areas. *G*. *duodenalis* is a species complex consisting of eight assemblages (A-H), with assemblages A and B the dominant assemblages in humans, C and D in canids, E in hoofed animals, F in felids, G in rodents and H in marine mammals [21]. In the present study, most (82.8%, 82/99) of the *Giardia*-positive samples by qPCR yielded C_T_ values ≥32, indicative of moderate-to-low parasite burdens. This fact explains the low amplification rates obtained at the *gdh*, *bg*, and *tpi* markers, all of them single-copy genes with limited sensitivity compared with the multiple-copy *ssu* rRNA gene used in qPCR for detection purposes. Our genotyping analyses revealed that assemblage A was more prevalent than assemblage B (50.0% *vs*. 37.5%). Similar frequencies (60% *vs*. 20%) have been previously identified in apparently healthy children in Pichincha province [37], but the opposite trend (32% *vs*. 61%) was observed in children with and without clinical manifestations in Esmeralda’s province [34]. Mixed A+B infections have been found in 7–12% of isolates (including this study), suggestive of epidemiological scenarios where infection/re-infection events are frequent. Overall, the vast majority of human cases of giardiasis characterised in Ecuador are due to assemblages A and B. This finding supports the notion that human *G*. *duodenalis* infections are primarily of anthropic nature. However, and unknown fraction of these infections might be of animal origin, as both assemblages A and B are zoonotic. Although probably infrequent, zoonotic transmission events are possible, as demonstrated by the occasional presence of canine-adapted assemblage C in two asymptomatic children in Pichincha province [37].

In the present study, *Blastocystis* sp. carriage rates were estimated at 39.2% by microscopy and at 22.6% by *ssu*-PCR. One explanation for this discrepancy could be an incorrect diagnosis, because microscopy detection and identification are challenging [63]. The epidemiology of *Blastocystis* sp. in Ecuador is largely unknown. The protist has been identified in 7–20% of asymptomatic individuals of all age groups both in urban and rural settings in Esmeraldas, Pichincha, and Santo Domingo de los Tsáchilas provinces by conventional microscopy, but a carriage rate as high as 81.5% was identified by PCR in similar populations in Esmeraldas and Manabí provinces (S2 Table). These data seem to suggest that *Blastocystis* sp. is ubiquitous in the Ecuadorian population. A total of 34 *Blastocystis* sp. subtypes (STs) are currently recognized, of which ST1-ST10, ST12, ST14, ST16, ST23, and ST35 have been reported in humans [20, 64]. ST1 (14.4%), ST2 (10.1%), ST3(19.2%), and ST4 (2.3%) are the most frequent *Blastocystis* sp. subtypes identified in humans in the Americas [65]. Interestingly, in the present study ST1-ST3 were found at very similar frequencies (27–30%), with ST4 being identified at a lower rate (15.0%). Of note, this is the first report of ST4 in Ecuador. In the only previous molecular epidemiological survey conducted in the country, ST1, ST2 and ST3 (alone or in combination) were identified in asymptomatic individuals in Esmeralda and Manabí provinces [39].

The fungi-related *E*. *bieneusi* is an emerging parasite responsible for 90% of human microsporidiosis reported worldwide [19]. It occurs most frequently in immunocompromised individuals [66], but it has also been reported in apparently healthy populations that might act as unnoticed disseminators [67]. More than 600 *E*. *bieneusi* genotypes distributed in 11 distinct phylogenetic groups have been described to date, which Group 1 and Group 2 comprising those with zoonotic potential [19]. Perhaps the most important contribution of this survey is the detection of *E*. *bieneusi* for the first time in Ecuador. Three different genotypes of *E*. *bieneusi* were identified, two known genotypes (A, KB-1) and a novel genotype (HhEcEb1). This finding demonstrated that an unexpected high diversity of *E*. *bieneusi* genetic variants were circulating in Ecuadorian asymptomatic children at low (but still significant) frequency rates. Remarkably, the identification of KB-1 represents also the second report of this genotype in humans globally after its initial description in a Chinese toddler attending kindergarten [68]. Previously, KB-1 was only described in a captive baboon in Kenya [69]. In contrast, the genotype A (syn. Peru1) of *E*. *bieneusi* is a common finding in human populations worldwide, particularly in immunocompromised patients [70], and has also been reported in other animals such non-human primates, dogs, or birds [19]. In a previous microscopy-based study conducted in Guayas province, microsporidial spores (of unknown species) were detected in 25% of HIV+ patients presenting with diarrhoea [35]. Our data contribute to improve our knowledge on the epidemiology of *E*. *bieneusi* in South America, a geographical region where this information is particularly scarce.

Previous studies conducted in Ecuador have reported the presence of members of the *Entamoeba* complex (*E*. *histolytica*/*E*. *dispar*/*E*. *moshkovskii*/*E*. *bangladeshi*) in 13–57% of the faecal samples examined by microscopy in different human populations (S2 Table). Because the transmission forms of these protists are morphologically indistinguishable, diagnostic differentiation of pathogenic *E*. *histolytica* and non-pathogenic *Entamoeba* species must be based on molecular methods. When PCR was used for this purpose, *E*. *histolytica* was detected at a prevalence rate of 2.8% (compared with the 69.8% of *E*. *dispar*) in rural and urban dwellers in Esmeraldas and Pichincha provinces [32]. In the present survey *E*. *histolytica* was not detected, whereas 14.5% of faecal DNA samples tested positive for *E*. *dispar* by qPCR.

Remarkably, no *Cryptosporidium* oocysts were detected using specific MZN staining. This apicomplexan protist has been identified at prevalence rates of 3–14% using microscopy as detection method in several Ecuadorian provinces including Azuay, Chimborazo, Esmeraldas, Pichincha, and Santo Domingo de los Tsáchilas provinces (S2 Table). As far as we know, no molecular-based studies have addressed the genetic diversity of *Cryptosporidium* spp. of human origin in Ecuador, this being a pending task that should be completed in future epidemiological surveys.

Our multivariate logistic regression analysis revealed marked differences of helminths and GP infection according to the municipality of origin, likely reflecting differences in socioeconomic status and sanitary conditions. When epidemiological data were stratified by municipality, variables associated with an increased risk of infection by intestinal parasite included overcrowded conditions (more than five people per household), limited or no access to safe drinking water, and inappropriate personal hygiene practices. These results are in line with those obtained in previous community surveys carried out in Ecuador [56, 59, 60]. Taken together, these findings demonstrate the strong link present between poverty and intestinal parasitic infections. Interventions directed to improve drinking water quality, household flooring and toilet facilities and personal hygiene practices are greatly needed to minimize helminths and GPs transmission to humans.

Several limitations might have compromised the accuracy of the results obtained and the conclusions reached in this study. First, epidemiological data generated here might not be representative of the whole Ecuadorian scenario; second, microscopy examination of a single stool sample per participating child was used as screening method, likely sub-estimating the true prevalence rates of helminths and GP and, in the case of the latter, reducing the chances of genotyping analyses; third, concentration devices involving centrifugation steps for the recovery of labile parasitic forms (e.g., trophozoites) might compromise the quality/quantity of template DNA used in PCR testing; fourth, the detection of *D*. *fragilis* trophozoites by direct microscopy would have required faecal smears stained with trichrome or another permanent stain and not, as in our case, directly on droplets of the sediment, therefore our microscopic data may be underestimating the real situation; fifth, insufficient number of participants in some locations might have compromised the robustness of the statistical analyses conducted, particularly when available data were stratified by municipality of origin; sixth, questionnaire data may have resulted in some recall/recording bias; and seventh, this surveys has focused on the human reservoir of helminths and GP, but the animal and environmental reservoirs have not been considered.

To conclude, this study shows that helminths and protists remain at high prevalence rates in Ecuadorian schoolchildren living in poor-resource settings, despite the MDA campaigns regularly conducted on this specific population. Our results indicate that albendazol mass administration, specifically designed to target STH, is insufficient to effectively control these pathogens and must be complemented with interventions directed to improve household quality and living conditions, sanitation, and health education for schoolchildren and families. Together with access to safe drinking water, these measures would contribute to the reduction of intestinal parasites in poor communities in Ecuador. This study also provided relevant molecular data, including the first description in Ecuadorian human populations of i) the genetic diversity of *E*. *bieneusi*, including a novel genotype, and ii) the presence of *Blastocystis* ST4, for the first time described in Ecuador.

Molecular analytical methods are therefore powerful complementary tools to microscopy, and essential to unravel the epidemiology of intestinal parasites (particularly GP) including sources of infection, transmission routes, zoonotic potential, and diagnostic differentiation between pathogenic and commensal species. More research should be conducted to ascertain the role of domestic and wild animals and the environment (source waters, soil, grass and fresh produce) in the epidemiology of STH and GP in endemic areas.

## Supporting information

S1 Table Occurrence of parasitic intestinal helminths in human populations, Ecuador, 2002–2022. (DOCX)

S2 Table Occurrence and molecular diversity of parasitic intestinal protists in human populations, Ecuador, 2002–2022. (DOCX)

S3 Table English version of the standardized epidemiological questionnaire used in the present study. (DOCX)

S4 Table Oligonucleotides used for the molecular identification and/or characterization of the parasitic intestinal protists. (DOCX)

S5 Table PCR cycling conditions used for the molecular identification and/or characterization of the parasitic intestinal protists. (DOCX)

S6 Table Multivariate logistic regression analysis for intestinal parasitism in the surveyed population according to the municipality of origin. Adjusted Odds Ratios (AORs) and 95% Confidential Intervals (95% CI) are indicated.
(DOCX)

## References

[1] Checkley W, White AC Jr, Jaganath D, Arrowood MJ, Chalmers RM, Chen XM, et al. A review of the global burden, novel diagnostics, therapeutics, and vaccine targets for *Cryptosporidium*. Lancet Infect Dis. 2015, 15(1): 85–94. doi: 10.1016/S1473-3099(14)70772-8 . 25278220 PMC4401121

[2] Kotloff KL. The burden and etiology of diarrheal illness in developing countries. Pediatr Clin North Am. 2017, 64(4): 799–814. doi: 10.1016/j.pcl.2017.03.006 . 28734511

[3] Montresor A, Mupfasoni D, Mikhailov A, Mwinzi P, Lucianez A, Jamsheed M, et al. The global progress of soil-transmitted helminthiases control in 2020 and World Health Organization targets for 2030. PLoS Negl Trop Dis. 2020, 14(8): e0008505. doi: 10.1371/journal.pntd.0008505 . 32776942 PMC7446869

[4] Petney TN, Andrews RH. Multiparasite communities in animals and humans: frequency, structure and pathogenic significance. Int J Parasitol. 1998, 28(3): 377–393. doi: 10.1016/s0020-7519(97)00189-6 . 9559357

[5] UNICEF. Malnutrition. 2021. https://data.unicef.org/topic/nutrition/malnutrition/#:~:text=In%202020*%2C%2022%20per%20cent,203.6%20million%20to%20149.2%20million. Accessed 12 January 2023.

[6] Black RE, Victora CG, Walker SP, Bhutta ZA, Christian P, de Onis M, et al. Maternal and child undernutrition and overweight in low-income and middle-income countries. Lancet. 2013; 382(9890): 427–451. doi: 10.1016/S0140-6736(13)60937-X . 23746772

[7] GBD 2019 Diseases and Injuries Collaborators. Global burden of 369 diseases and injuries in 204 countries and territories, 1990–2019: a systematic analysis for the Global Burden of Disease Study 2019. Lancet. 2020; 396(10258): 1204–1222. doi: 10.1016/S0140-6736(20)30925-9 . 33069326 PMC7567026

[8] WHO. Neglected tropical diseases. 2023. https://www.who.int/news-room/questions-and-answers/item/neglected-tropical-diseases. Accessed 3 March 2023.

[9] Bethony J, Brooker S, Albonico M, Geiger SM, Loukas A, Diemert D, et al. Soil-transmitted helminth infections: ascariasis, trichuriasis, and hookworm. Lancet. 2006; 367(9521): 1521–1532. doi: 10.1016/S0140-6736(06)68653-4 . 16679166

[10] Guideline: Preventive chemotherapy to control soil-transmitted helminth infections in at-risk population groups. Geneva: World Health Organization; 2017. 29578660

[11] Fleitas PE, Travacio M, Martí-Soler H, Socías ME, Lopez WR, Krolewiecki AJ. The *Strongyloides stercoralis*-hookworms association as a path to the estimation of the global burden of strongyloidiasis: A systematic review. PLoS Negl Trop Dis. 2020; 14(4): e0008184. doi: 10.1371/journal.pntd.0008184 . 32282827 PMC7188296

[12] Prüss-Ustün A, Wolf J, Bartram J, Clasen T, Cumming O, Freeman MC, et al. Burden of disease from inadequate water, sanitation and hygiene for selected adverse health outcomes: An updated analysis with a focus on low- and middle-income countries. Int J Hyg Environ Health. 2019; 222(5): 765–777. doi: 10.1016/j.ijheh.2019.05.004 . 31088724 PMC6593152

[13] Kotloff KL, Nataro JP, Blackwelder WC, Nasrin D, Farag TH, Panchalingam S, et al. Burden and aetiology of diarrhoeal disease in infants and young children in developing countries (the Global Enteric Multicenter Study, GEMS): a prospective, case-control study. Lancet. 2013; 382(9888): 209–222. doi: 10.1016/S0140-6736(13)60844-2 . 23680352

[14] Murray CJ, Vos T, Lozano R, Naghavi M, Flaxman AD, Michaud C, et al. Disability-adjusted life years (DALYs) for 291 diseases and injuries in 21 regions, 1990–2010: a systematic analysis for the Global Burden of Disease Study 2010. Lancet. 2012; 380 (9859): 2197–2223. doi: 10.1016/S0140-6736(12)61689-4 . 23245608

[15] WHO. The World Health Report. Fighting disease fostering development. Geneva: Switzerland; 1996. http://www.who.int/whr/1996/en/. Accessed 12 January 2023.

[16] Levine MM, Nasrin D, Acácio S, Bassat Q, Powell H, Tennant SM, et al. Diarrhoeal disease and subsequent risk of death in infants and children residing in low-income and middle-income countries: analysis of the GEMS case-control study and 12-month GEMS-1A follow-on study. Lancet Glob Health. 2020; 8(2): e204–214. doi: 10.1016/S2214-109X(19)30541-8 . 31864916 PMC7025325

[17] Li W, Feng Y, Xiao L. Enterocytozoon bieneusi. Trends Parasitol. 2022; 38(1):95–96. doi: 10.1016/j.pt.2021.08.003 34474945

[18] Andersen LO, Stensvold CR. *Blastocystis* in health and disease: Are we moving from a clinical to a public health perspective? J Clin Microbiol. 2016; 54(3): 524–528. doi: 10.1128/JCM.02520-15 . 26677249 PMC4767957

[19] Li W, Feng Y, Santin M. Host specificity of *Enterocytozoon bieneusi* and public health implications. Trends Parasitol. 2019; 35(6): 436–451. doi: 10.1016/j.pt.2019.04.004 . 31076351

[20] Stensvold CR, Clark CG. Pre-empting Pandora’s box: *Blastocystis* subtypes revisited. Trends Parasitol. 2020; 36(3): 229–232. doi: 10.1016/j.pt.2019.12.009 . 32001133

[21] Ryan UM, Feng Y, Fayer R, Xiao L. Taxonomy and molecular epidemiology of *Cryptosporidium* and *Giardia*—a 50 year perspective (1971–2021). Int J Parasitol. 2021; 51(13–14): 1099–1119. doi: 10.1016/j.ijpara.2021.08.007 . 34715087

[22] Bartram J, Cairncross S. Hygiene, sanitation, and water: forgotten foundations of health. PLoS Med. 2010; 7(11): e1000367. doi: 10.1371/journal.pmed.1000367 . 21085694 PMC2976722

[23] Garn JV, Wilkers JL, Meehan AA, Pfadenhauer LM, Burns J, Imtiaz R, et al. Interventions to improve water, sanitation, and hygiene for preventing soil-transmitted helminth infection. Cochrane Database Syst Rev. 2022; 6(6): CD012199. doi: 10.1002/14651858.CD012199.pub2 . 35726112 PMC9208960

[24] Wolf J, Hubbard S, Brauer M, Ambelu A, Arnold BF, Bain R, et al. Effectiveness of interventions to improve drinking water, sanitation, and handwashing with soap on risk of diarrhoeal disease in children in low-income and middle-income settings: a systematic review and meta-analysis. Lancet. 2022, 400(10345): 48–59. doi: 10.1016/S0140-6736(22)00937-0 . 35780792 PMC9251635

[25] Jacobsen KH, Ribeiro PS, Quist BK, Rydbeck BV. Prevalence of intestinal parasites in young Quichua children in the highlands of rural Ecuador. J Health Popul Nutr. 2007; 25(4): 399–405 . 18402182 PMC2754013

[26] Mejia R, Vicuña Y, Broncano N, Sandoval C, Vaca M, Chico M, et al. A novel, multi-parallel, real-time polymerase chain reaction approach for eight gastrointestinal parasites provides improved diagnostic capabilities to resource-limited at-risk populations. Am J Trop Med Hyg. 2013; 88(6): 1041–1047. doi: 10.4269/ajtmh.12-0726 . 23509117 PMC3752800

[27] Weatherhead J, Cortés AA, Sandoval C, Vaca M, Chico M, Loor S, et al. Comparison of cytokine responses in Ecuadorian children infected with *Giardia*, *Ascaris*, or both parasites. Am J Trop Med Hyg. 2017; 96(6): 1394–1399. doi: 10.4269/ajtmh.16-0580 . 28719267 PMC5462578

[28] Abad-Sojos G, Gómez-Barreno L, Inga-Salazar G, Simbaña-Pilataxi D, Flores-Enríquez J, Martínez-Cornejo I, et al. Presencia de parasitosis intestinal en una comunidad escolar urbano marginal del Ecuador. CIMEL 2017; 22(2): 52. 10.23961/cimel.v22i2.953.

[29] Sackey ME, Weigel MM, Armijos RX. Predictors and nutritional consequences of intestinal parasitic infections in rural Ecuadorian children. J Trop Pediatr. 2003; 49(1): 17–23. doi: 10.1093/tropej/49.1.17 . 12630715

[30] Calvopina M, Atherton R, Romero-Álvarez D, Castaneda B, Valverde-Muñoz G, Cevallos W, et al. Identification of intestinal parasite infections and associated risk factors in indigenous Tsáchilas communities of Ecuador. Int J Acad Med. 2019; 5(3): 171–179.

[31] Levecke B, Dreesen L, Barrionuevo-Samaniego M, Ortiz WB, Praet N, Brandt J, et al. Molecular differentiation of *Entamoeba* spp. in a rural community of Loja province, South Ecuador. Trans R Soc Trop Med Hyg. 2011; 105(12): 737–739. doi: 10.1016/j.trstmh.2011.08.010 . 21981992

[32] Guevara Á, Vicuña Y, Costales D, Vivero S, Anselmi M, Bisoffi Z, et al. Use of real-time polymerase chain reaction to differentiate between pathogenic *Entamoeba histolytica* and the nonpathogenic *Entamoeba dispar* in Ecuador. Am J Trop Med Hyg. 2019; 100(1): 81–82. doi: 10.4269/ajtmh.17-1022 . 30398142 PMC6335901

[33] Palacios Ordóñez TE. Prevalencia de *Cryptosporidium* spp. y *Giardia* spp. en terneros, y su presencia en agua y en niños con problemas digestivos en el cantón San Fernando, Ecuador. MASKANA. 2017; 8(1): 111–119. 10.18537/mskn.08.01.10 111

[34] Atherton R, Bhavnani D, Calvopiña M, Vicuña Y, Cevallos W, Eisenberg J. Molecular identification of *Giardia duodenalis* in Ecuador by polymerase chain reaction-restriction fragment length polymorphism. Mem Inst Oswaldo Cruz. 2013; 108(4):512–515. doi: 10.1590/S0074-02762013000400019 . 23827993 PMC3970629

[35] Pazmiño B, Rodas E, Rodas J, Zambrano R, Dávila A, Martini L, Pazmiño CA, Día L. Microsporidium spp. en pacientes VIH positivos con síndrome diarreico. REVISTA. 2014; 17(2): 14–21.

[36] Vasco K, Graham JP, Trueba G. Detection of zoonotic enteropathogens in children and domestic animals in a semirural community in Ecuador. Appl Environ Microbiol. 2016; 82(14): 4218–4224. doi: 10.1128/AEM.00795-16 . 27208122 PMC4959199

[37] Sarzosa M, Graham JP, Salinas L, Trueba G. Potential zoonotic transmission of *Giardia duodenalis* in semi-rural communities near Quito, Ecuador. Intern J Appl Res Vet Med. 2018; 16(1):1–6.

[38] Cajamarca A, Criollo D, Solano R, Sacoto A, Mosquera L. Estudio experimental: prevención de parasitosis en escolares de una zona rural. Azuay, Ecuador. 2013–2014. Rev Med HJCA. 2017; 9(2): 139–143. doi: 10.14410/2017.9.2.ao.23

[39] Helenbrook WD, Shields WM, Whipps CM. Characterization of *Blastocystis* species infection in humans and mantled howler monkeys, *Alouatta palliata aequatorialis*, living in close proximity to one another. Parasitol Res. 2015; 114(7): 2517–2525. doi: 10.1007/s00436-015-4451-x . 25859926

[40] Lowenstein C, Vasco K, Sarzosa S, Salinas L, Torres A, Perry MJ, et al. Determinants of childhood zoonotic enteric infections in a semirural community of Quito, Ecuador. Am J Trop Med Hyg. 2020; 102(6): 1269–1278. doi: 10.4269/ajtmh.19-0690 . 32228797 PMC7253092

[41] Instituto Nacional de Estadística y Censos (INEC). Censo de Población y Viviendo 2010. Available online: https://urldefense.com/v3/__ http://app.sni.gob.ec/sni-link/sni/Portal*20SNI*202014/FICHAS*20F/0608_PALLATANGA_CHIMBORAZO.pdf__;JSUl!!D9dNQwwGXtA!Q893ljJga3GQU5oOpiljJsOpYLz4Jm6Iapaq95yYnu3-1YL_goR2th_NcGJvXHeGsJ3is93as5OkIowPgwaJ$. Accessed 12 January 2023.

[42] Instituto Nacional de Estadística y Censos (INEC). Censo de Población y Viviendo 2010. Available online: https://urldefense.com/v3/__https://www.ecuadorencifras.gob.ec/documentos/webinec/Poblacion_y_Demografia/CPV_aplicativos/datos_generales_cpv/09generalantonioelizalde.pdf__;!!D9dNQwwGXtA!Q893ljJga3GQU5oOpiljJsOpYLz4Jm6Iapaq95yYnu3-1YL_goR2th_NcGJvXHeGsJ3is93as5OkIsHXpbz9$. Accessed 12 January 2023.

[43] Villacís B, Carrillo D. País atrevido: la nueva cara sociodemográfica del Ecuador, edición especial revista analítica; Instituto Nacional de Estadística y Censos (INEC). Quito, Ecuador. 2012. Available online: https://urldefense.com/v3/__https://www.ecuadorencifras.gob.ec/wp-content/descargas/Libros/Economia/Nuevacarademograficadeecuador.pdf__;!!D9dNQwwGXtA!Q893ljJga3GQU5oOpiljJsOpYLz4Jm6Iapaq95yYnu3-1YL_goR2th_NcGJvXHeGsJ3is93as5OkImVpZThV$. Accessed 12 January 2023.

[44] Verweij JJ, Schinkel J, Laeijendecker D, van Rooyen MA, van Lieshout L, Polderman AM. Real-time PCR for the detection of *Giardia lamblia*. Mol Cell Probes. 2003; 17(5): 223–225. doi: 10.1016/s0890-8508(03)00057-4 . 14580396

[45] Read CM, Monis PT, Thompson RC. Discrimination of all genotypes of *Giardia duodenalis* at the glutamate dehydrogenase locus using PCR-RFLP. Infect Genet Evol. 2004; 4(2): 125–130. doi: 10.1016/j.meegid.2004.02.001 . 15157630

[46] Lalle M, Pozio E, Capelli G, Bruschi F, Crotti D, Cacciò SM. Genetic heterogeneity at the beta-giardin locus among human and animal isolates of *Giardia duodenalis* and identification of potentially zoonotic subgenotypes. Int J Parasitol. 2005; 35(2): 207–13. doi: 10.1016/j.ijpara.2004.10.022 . 15710441

[47] Sulaiman IM, Fayer R, Bern C, Gilman RH, Trout JM, Schantz PM, et al. Triosephosphate isomerase gene characterization and potential zoonotic transmission of *Giardia duodenalis*. Emerg Infect Dis. 2003; 9(11): 1444–1452. doi: 10.3201/eid0911.030084 . 14718089 PMC3035538

[48] Gutiérrez-Cisneros MJ, Cogollos R, López-Vélez R, Martín-Rabadán P, Martínez-Ruiz R, Subirats M, et al. Application of real-time PCR for the differentiation of *Entamoeba histolytica* and *E*. *dispar* in cyst-positive faecal samples from 130 immigrants living in Spain. Ann Trop Med Parasitol. 2010; 104(2): 145–149. doi: 10.1179/136485910X12607012373759 . 20406581

[49] Verweij JJ, Oostvogel F, Brienen EA, Nang-Beifubah A, Ziem J, Polderman AM. Prevalence of *Entamoeba histolytica* and *Entamoeba dispar* in northern Ghana. Trop Med Int Health. 2003; 8(12): 1153–1156. doi: 10.1046/j.1360-2276.2003.01145.x . 14641852

[50] Tiangtip R, Jongwutiwes S. Molecular analysis of *Cryptosporidium* species isolated from HIV-infected patients in Thailand. Trop Med Int Health. 2002; 7(4): 357–364. doi: 10.1046/j.1365-3156.2002.00855.x . 11952952

[51] Scicluna SM, Tawari B, Clark CG. DNA barcoding of *Blastocystis*. Protist. 2006; 157(1): 77–85. doi: 10.1016/j.protis.2005.12.001 . 16431158

[52] Buckholt MA, Lee JH, Tzipori S. Prevalence of *Enterocytozoon bieneusi* in swine: an 18-month survey at a slaughterhouse in Massachusetts. Appl Environ Microbiol. 2002; 68(5): 2595–2599. doi: 10.1128/AEM.68.5.2595–2599.2002 11976142 PMC127518

[53] Kumar S, Stecher G, Li M, Knyaz C, Tamura K. MEGA X: Molecular Evolutionary Genetics Analysis across Computing Platforms. Mol Biol Evol. 2018; 35(6): 1547–1549. doi: 10.1093/molbev/msy096 . 29722887 PMC5967553

[54] Saitou N, Nei M. The neighbor-joining method: a new method for reconstructing phylogenetic trees. Mol Biol Evol. 1987;4: 406–425. doi: 10.1093/oxfordjournals.molbev.a040454 . 3447015

[55] Graham CF. A device for the diagnosis of *Enterobius* infection. Am J Trop Med Hyg. 1941; 1941: 159–161. doi: 10.4269/ajtmh.1941.s1-21.159

[56] Chis Ster I, Niaz HF, Chico ME, Oviedo Y, Vaca M, Cooper PJ. The epidemiology of soil-transmitted helminth infections in children up to 8 years of age: Findings from an Ecuadorian birth cohort. PLoS Negl Trop Dis. 2021; 15(11): e0009972. doi: 10.1371/journal.pntd.0009972 . 34797823 PMC8641893

[57] Moncayo AL, Lovato R, Cooper PJ. Soil-transmitted helminth infections and nutritional status in Ecuador: findings from a national survey and implications for control strategies. BMJ Open. 2018; 8(4): e021319. doi: 10.1136/bmjopen-2017-021319 . 29705768 PMC5931300

[58] Moncayo AL, Granizo G, Grijalva MJ, Rasella D. Strong effect of Ecuador’s conditional cash transfer program on childhood mortality from poverty-related diseases: a nationwide analysis. BMC Public Health. 2019; 19(1): 1132. doi: 10.1186/s12889-019-7457-y . 31420035 PMC6697994

[59] Gildner TE, Cepon-Robins TJ, Liebert MA, Urlacher SS, Madimenos FC, Snodgrass JJ, et al. Regional variation in *Ascaris lumbricoides* and *Trichuris trichiura* infections by age cohort and sex: effects of market integration among the indigenous Shuar of Amazonian Ecuador. J Physiol Anthropol. 2016; 35(1): 28. doi: 10.1186/s40101-016-0118-2 . 27884213 PMC5123216

[60] Gildner TE, Cepon-Robins TJ, Liebert MA, Urlacher SS, Schrock JM, Harrington CJ, et al. Market integration and soil-transmitted helminth infection among the Shuar of Amazonian Ecuador. PLoS One. 2020; 15(7): e0236924. doi: 10.1371/journal.pone.0236924 . 32735608 PMC7394393

[61] Mejia R, Seco-Hidalgo V, Garcia-Ramon D, Calderón E, Lopez A, Cooper PJ. Detection of enteric parasite DNA in household and bed dust samples: potential for infection transmission. Parasit Vectors. 2020; 13(1): 141. doi: 10.1186/s13071-020-04012-6 . 32188497 PMC7079405

[62] Sarria-Guzmán Y, Chávez-Romero Y, Bernal JE, González-Jiménez FE, Serrano-Silva N, Fusaro C. Molecular identification of *Giardia* spp. in Latin America: An updated systematic review on reports from 2017 to 2021. J Infect Dev Ctries. 2022; 16(3): 392–401. doi: 10.3855/jidc.15806 . 35404842

[63] Roberts T, Barratt J, Harkness J, Ellis J, Stark D. Comparison of microscopy, culture, and conventional polymerase chain reaction for detection of *Blastocystis* sp. in clinical stool samples. Am J Trop Med Hyg. 2011;; 84(2): 308–312. doi: 10.4269/ajtmh.2011.10–0447 . 21292905 PMC3029188

[64] Maloney JG, Molokin A, Seguí R, Maravilla P, Martínez-Hernández F, Villalobos G, et al. Identification and molecular characterization of four new *Blastocystis* subtypes designated ST35-ST38. Microorganisms. 2022; 23;11(1):46. doi: 10.3390/microorganisms11010046 ; PMCID: PMC9860945. 36677338 PMC9860945

[65] Jiménez P, Muñoz M, Ramírez JD. An update on the distribution of *Blastocystis* subtypes in the Americas. Heliyon. 2022; 8(12): e12592. doi: 10.1016/j.heliyon.2022.e12592 . 36619449 PMC9816782

[66] Lobo ML, Xiao L, Antunes F, Matos O. Microsporidia as emerging pathogens and the implication for public health: a 10-year study on HIV-positive and -negative patients. Int J Parasitol. 2012; 42(2): 197–205. doi: 10.1016/j.ijpara.2011.12.002 . 22265899

[67] Sak B, Brady D, Pelikánová M, Květoňová D, Rost M, Kostka M, et al. Unapparent microsporidial infection among immunocompetent humans in the Czech Republic. J Clin Microbiol. 2011; 49(3): 1064–1070. doi: 10.1128/JCM.01147-10 . 21191056 PMC3067711

[68] Qi M, Yu F, Zhao A, Zhang Y, Wei Z, Li D, et al. Unusual dominant genotype NIA1 of *Enterocytozoon bieneusi* in children in Southern Xinjiang, China. PLoS Negl Trop Dis. 2020; 14(6): e0008293. doi: 10.1371/journal.pntd.0008293 . 32569279 PMC7332067

[69] Li W, Kiulia NM, Mwenda JM, Nyachieo A, Taylor MB, Zhang X, et al. *Cyclospora papionis*, *Cryptosporidium hominis*, and human-pathogenic *Enterocytozoon bieneusi* in captive baboons in Kenya. J Clin Microbiol. 2011; 49(12):4326–4329. doi: 10.1128/JCM.05051-11 . 21956988 PMC3232936

[70] Sulaiman IM, Bern C, Gilman R, Cama V, Kawai V, Vargas D, et al. A molecular biologic study of *Enterocytozoon bieneusi* in HIV-infected patients in Lima, Peru. J Eukaryot Microbiol. 2003; 50:591–596. doi: 10.1111/j.1550-7408.2003.tb00642.x 14736175

