# Supplementary material for: Prevalence and associated risk factors of intestinal parasites among schoolchildren in Ecuador, with emphasis on the molecular diversity of Giardia duodenalis, Blastocystis sp. and Enterocytozoon bieneusi
Source: PLoS Negl Trop Dis. 2023 May 24;17(5):e0011339. doi: 10.1371/journal.pntd.0011339 (PMC10243618; doi:10.1371/journal.pntd.0011339)
Supplement: S1 Table — (DOCX) [file pntd.0011339.s001.docx]

**Table S1.** Occurrence of parasitic intestinal helminths in human populations, Ecuador, 2002-2022.

| **Population** | **Province** | **Detection method** | **Samples (*n*)** | **Parasite species** | **Infection rate (%)** | **References** |
| --- | --- | --- | --- | --- | --- | --- |
| Asymptomatic children | Chimborazo | CM | 203 | *Ascaris lumbricoides* | 35.5 | [1] |
|  |  |  |  | *Hymenolepis nana* | 11.3 |  |
|  |  |  |  | *Hymenolepis diminuta* | 1.0 |  |
|  |  |  |  | *Strongyloides stercoralis* | 0.7 |  |
|  |  |  |  | *Trichuris trichiura* | 0.5 |  |
| Asymptomatic children | Esmeraldas | CM, qPCR | 400 | *Ascaris lumbricoides* | 7.0 | [2] |
|  |  |  |  | *Trichuris trichiura* | 3.0 |  |
|  |  |  |  | *Strongyloides stercoralis* | 0.8 |  |
|  |  |  |  | *Ancylostoma duodenale* | 0.5 |  |
| Asymptomatic children | Esmeraldas | ELISA | 39 | *Ascaris lumbricoides* | 17.9 | [3] |
| Asymptomatic children | Manabí | CM | 112 | *Ascaris lumbricoides* | 9.8 | [4] |
|  |  |  |  | *Hymenolepis nana* | 3.3 |  |
| Asymptomatic children | Pichincha | CM | 244 | *Ascaris lumbricoides* | 39.7 | [5] |
|  |  |  |  | *Trichuris trichiura* | 19.7 |  |
| Asymptomatic (all age groups) | St. Domingo de los Tsáchilas | CM | 586 | *Ascaris lumbricoides* | 29.4 | [6] |
|  |  |  |  | *Trichuris trichiura* | 11.4 |  |
|  |  |  |  | *Hookworm* | 1.0 |  |
|  |  |  |  | *Amphimerus* sp. | 0.5 |  |
|  |  |  |  | *Enterobius vermicularis* | 0.5 |  |
|  |  |  |  | *Hymenolepis* | 0.2 |  |
|  |  |  |  | *Paragonimus westermani* | 0.3 |  |
|  |  |  |  | *Strongyloides stercoralis* | 0.5 |  |
| Rural dwellers | Loja | CM | 674 | *Hymenolepis nana* | 4.3 | [7] |
|  |  |  |  | *Strongyloides stercoralis* | 4.3 |  |
|  |  |  |  | *Trichuris trichiura* | 4.3 |  |
| Rural and urban dwellers | Esmeraldas, Pichincha | CM, PCR | 106 | *Ascaris lumbricoides* | 28.9–45.2 | [8] |
|  |  |  |  | *Trichuris trichiura* | 10.9–24.5 |  |
|  |  |  |  | *Enterobius vermicularis* | 10.6 |  |
|  |  |  |  | *Strongyloides stercoralis* | 3.8–8.2 |  |
|  |  |  |  | *Hymenolepis nana* | 6.3 |  |
|  |  |  |  | *Taenia* spp. | 2.0 |  |

CM: Conventional microscopy; ELISA: Enzyme-Linked Immunosorbent Assay; PCR: Polymerase Chain Reaction; qPCR: real-time PCR.

**References**

1. Jacobsen KH, Ribeiro PS, Quist BK, Rydbeck BV. Prevalence of intestinal parasites in young Quichua children in the highlands of rural Ecuador. J Health Popul Nutr. 2007; 25(4): 399–405 PMID: 18402182.
2. Mejia R, Vicuña Y, Broncano N, Sandoval C, Vaca M, Chico M, et al. A novel, multi-parallel, real-time polymerase chain reaction approach for eight gastrointestinal parasites provides improved diagnostic capabilities to resource-limited at-risk populations. Am J Trop Med Hyg. 2013; 88(6): 1041–1047. doi: 10.4269/ajtmh.12-0726 PMID: 23509117.
3. Weatherhead J, Cortés AA, Sandoval C, Vaca M, Chico M, Loor S, et al. Comparison of cytokine responses in Ecuadorian children infected with *Giardia*, *Ascaris*, or both parasites. Am J Trop Med Hyg. 2017; 96(6): 1394–1399. doi: 10.4269/ajtmh.16-0580 PMID: 28719267.
4. Abad-Sojos G, Gómez-Barreno L, Inga-Salazar G, Simbaña-Pilataxi D, Flores-Enríquez J, Martínez-Cornejo I, et al. Presencia de parasitosis intestinal en una comunidad escolar urbano marginal del Ecuador. CIMEL 2017; 22(2): 52. doi: https://doi.org/10.23961/cimel.v22i2.953.
5. Sackey ME, Weigel MM, Armijos RX. Predictors and nutritional consequences of intestinal parasitic infections in rural Ecuadorian children. J Trop Pediatr. 2003; 49(1): 17–23. doi: 10.1093/tropej/49.1.17 PMID: 12630715.
6. Calvopina M, Atherton R, Romero-Álvarez D, Castaneda B, Valverde-Muñoz G, Cevallos W, Izurieta R. Identification of intestinal parasite infections and associated risk factors in indigenous Tsáchilas communities of Ecuador. Int J Acad Med. 2019; 5(3): 171–179.
7. Levecke B, Dreesen L, Barrionuevo-Samaniego M, Ortiz WB, Praet N, Brandt J, et al. Molecular differentiation of *Entamoeba* spp. in a rural community of Loja province, South Ecuador. Trans R Soc Trop Med Hyg. 2011; 105(12): 737–739. doi: 10.1016/j.trstmh.2011.08.010 PMID: 21981992.
8. Guevara Á, Vicuña Y, Costales D, Vivero S, Anselmi M, Bisoffi Z, et al. Use of real-time polymerase chain reaction to differentiate between pathogenic *Entamoeba histolytica* and the nonpathogenic *Entamoeba dispar* in Ecuador. Am J Trop Med Hyg. 2019; 100(1): 81–82. doi: 10.4269/ajtmh.17-1022 PMID: 30398142.
