# Supplementary material for: Prevalence and associated risk factors of intestinal parasites among schoolchildren in Ecuador, with emphasis on the molecular diversity of Giardia duodenalis, Blastocystis sp. and Enterocytozoon bieneusi
Source: PLoS Negl Trop Dis. 2023 May 24;17(5):e0011339. doi: 10.1371/journal.pntd.0011339 (PMC10243618; doi:10.1371/journal.pntd.0011339)
Supplement: S2 Table — (DOCX) [file pntd.0011339.s002.docx]

**Table S2.** Occurrence and molecular diversity of parasitic intestinal protists in human populations, Ecuador, 2002-2022.

| **Population** | **Province** | **Detection method** | **Samples (*n*)** | **Parasite species** | **Infection rate (%)** | **Genotype (*n*)** | **References** |
| --- | --- | --- | --- | --- | --- | --- | --- |
| Symptomatic children | Azuay | CM | 42 | *Giardia duodenalis* | 33.3 | ND | [1] |
|  |  |  |  | *Cryptosporidium* spp. | 14.3 | ND |  |
| Symptomatic and asymptomatic children | Esmeraldas | ELISA, PCR-RLFP | 592 | *Giardia duodenalis* | 26.0 | AI (3), AII (19), BIII (26), BIV (16), AII+BIII (5) | [2] |
| HIV+ patients with diarrhoea | Guayas | CM, DFA, SEM | 89 | Microsporidia | 25.0 | ND | [3] |
| Asymptomatic children | Chimborazo | CM | 203 | *Entamoeba* complex^a^ | 57.1 | ND | [4] |
|  |  |  |  | *Entamoeba coli* | 34.0 | ND |  |
|  |  |  |  | *Giardia duodenalis* | 21.1 | ND |  |
|  |  |  |  | *Cryptosporidium* spp. | 8.9 | ND |  |
|  |  |  |  | *Chilomastix mesnili* | 1.7 | ND |  |
| Asymptomatic children | Esmeraldas | CM, qPCR | 400 | *Giardia duodenalis* | 31.5 | ND | [5] |
|  |  |  |  | *Cryptosporidium* spp. | 5.3 | ND |  |
|  |  |  |  | *Entamoeba. histolytica* | 1.0 | ND |  |
| Asymptomatic children | Esmeraldas | ELISA | 39 | *Giardia duodenalis* | 28.2 | ND | [6] |
| Asymptomatic children | Manabí | CM | 112 | *Entamoeba coli* | 36.0 | ND | [7] |
|  |  |  |  | *Entamoeba* complex^a^ | 34.4 | ND |  |
|  |  |  |  | *Giardia duodenalis* | 16.4 | ND |  |
| Asymptomatic children | Pichincha | CM | 244 | *Giardia duodenalis* | 39.7 | ND | [8] |
|  |  |  |  | *Entamoeba* complex^a^ | 18.5 | ND |  |
| Asymptomatic children | Pichincha | ELISA | 64 | *Giardia duodenalis* | 34.4 | ND | [9] |
|  |  |  |  | *Cryptosporidium* spp. | 3.1 | ND |  |
| Asymptomatic children | Pichincha | ELISA, PCR | 316 | *Giardia duodenalis* | 20.0 | A (6), B (2), C (2) | [10] |
| Asymptomatic  (all age groups) | Azuay | CM | 335 | NS^b^ | 46.2 | ND | [11] |
| Asymptomatic  (all age groups) | Esmeraldas, Manabí | PCR-SSCP | 55 | *Blastocystis sp.* | 81.5 | ST1 (21), ST2 (10), ST3 (12), ST1+ST3 (1), ST1+ST2 (2) | [12] |
| Asymptomatic  (all age groups) | St. Domingo de los Tsáchilas | CM | 586 | *Entamoeba coli* | 27.5 | ND | [13] |
|  |  |  |  | *Blastocystis* sp. | 19.6 |  |  |
|  |  |  |  | *Entamoeba* complex^a^ | 12.5 | ND |  |
|  |  |  |  | *Entamoeba hartmanni* | 10.8 |  |  |
|  |  |  |  | *Endolimax nana* | 4.9 |  |  |
|  |  |  |  | *Giardia duodenalis* | 3.9 | ND |  |
|  |  |  |  | *Iodamoeba butschlii* | 3.6 |  |  |
|  |  |  |  | *Chilomastix mesnili* | 1.4 | ND |  |
| Asymptomatic  (all age groups) | St. Domingo de los Tsáchilas | ELISA | 306 | *Giardia duodenalis* | 20.3 | ND | [14] |
|  |  |  |  | *Cryptosporidium* spp. | 4.3 | ND |  |
| Rural dwellers | Loja | CM, PCR-RLHB^c^ | 674 | *Endolimax nana* | 47.0 |  | [15] |
|  |  |  |  | *Entamoeba coli* | 28.0 | ND |  |
|  |  |  |  | *Entamoeba* complex^a^ | 16.2 | ND |  |
|  |  |  |  | *Chilomastix mesnili* | <4.3 | ND |  |
|  |  |  |  | *Giardia duodenalis* | <4.3 | ND |  |
|  |  |  |  | *Iodamoeba bütschlii* | <4.3 | ND |  |
| Rural and urban dwellers | Esmeraldas, Pichincha | CM, PCR^d^ | 106 | *Entamoeba dispar* | 69.8 | ND | [16] |
|  |  |  |  | *Entamoeba coli* | 60.4 | ND |  |
|  |  |  |  | *Giardia duodenalis* | 10.4 | ND |  |
|  |  |  |  | *Blastocystis* sp. | 6.6 | ND |  |
|  |  |  |  | *Enbadomonas duodenalis* | 6.6 | ND |  |
|  |  |  |  | *Enteromonas duodenalis* | 5.6 | ND |  |
|  |  |  |  | *Iodamoeba butschlii* | 5.6 | ND |  |
|  |  |  |  | *Endolimax nana* | 3.8 | ND |  |
|  |  |  |  | *Entamoeba histolytica* | 2.8 | ND |  |

CM: Conventional microscopy; DFA: Direct Fluorescent Antibody assay; ELISA: Enzyme-Linked Immunosorbent Assay; ND: Not Determined; NS: Not Specified; PCR: Polymerase Chain Reaction; PCR-RFLP: Polymerase Chain Reaction-restriction Fragment Length Polymorphism; PCR-SSCP: Polymerase Chain Reaction-Single Strand Conformation Polymorphism, PCR-RLHB: Polymerase Chain Reaction-Reverse Line Hybridization Blot; SEM: Scanning Electron Microscopy.

^a^ *Entamoeba* complex: *Entamoeba histolytica*/*Entamoeba dispar*/*Entamoeba moshkovskii/ E.* *bangladeshi*

^b^ Study reporting general infection rates by any intestinal parasite.

^c^ Only for the differential detection of members of the *Entamoeba* complex.

^d^ Only for the differential detection of *E. histolytica* and *E. dispar*.

**References**

1. Palacios Ordóñez TE. Prevalencia de *Cryptosporidium* spp. y *Giardia* spp. en terneros, y su presencia en agua y en niños con problemas digestivos en el cantón San Fernando, Ecuador. MASKANA. 2017; 8(1): 111–119. https://doi.org/10.18537/mskn.08.01.10 111
2. Atherton R, Bhavnani D, Calvopiña M, Vicuña Y, Cevallos W, Eisenberg J. Molecular identification of *Giardia duodenalis* in Ecuador by polymerase chain reaction-restriction fragment length polymorphism. Mem Inst Oswaldo Cruz. 2013; 108(4):512–515. doi: 10.1590/S0074-02762013000400019 PMID: 23827993.
3. Pazmiño B, Rodas E, Rodas J, Zambrano R, Dávila A, Martini L, Pazmiño CA, Día L. Microsporidium spp. en pacientes VIH positivos con síndrome diarreico. REVISTA. 2014; 17(2): 14–21.
4. Jacobsen KH, Ribeiro PS, Quist BK, Rydbeck BV. Prevalence of intestinal parasites in young Quichua children in the highlands of rural Ecuador. J Health Popul Nutr. 2007; 25(4): 399–405 PMID: 18402182.
5. Mejia R, Vicuña Y, Broncano N, Sandoval C, Vaca M, Chico M, et al. A novel, multi-parallel, real-time polymerase chain reaction approach for eight gastrointestinal parasites provides improved diagnostic capabilities to resource-limited at-risk populations. Am J Trop Med Hyg. 2013; 88(6): 1041–1047. doi: 10.4269/ajtmh.12-0726 PMID: 23509117.
6. Weatherhead J, Cortés AA, Sandoval C, Vaca M, Chico M, Loor S, et al. Comparison of cytokine responses in Ecuadorian children infected with Giardia, Ascaris, or both parasites. Am J Trop Med Hyg. 2017; 96(6): 1394–1399. doi: 10.4269/ajtmh.16-0580 PMID: 28719267.
7. Abad-Sojos G, Gómez-Barreno L, Inga-Salazar G, Simbaña-Pilataxi D, Flores-Enríquez J, Martínez-Cornejo I, et al. Presencia de parasitosis intestinal en una comunidad escolar urbano marginal del Ecuador. CIMEL 2017; 22(2): 52. doi: https://doi.org/10.23961/cimel.v22i2.953.
8. Sackey ME, Weigel MM, Armijos RX. Predictors and nutritional consequences of intestinal parasitic infections in rural Ecuadorian children. J Trop Pediatr. 2003; 49(1): 17–23. doi: 10.1093/tropej/49.1.17 PMID: 12630715.
9. Vasco K, Graham JP, Trueba G. Detection of zoonotic enteropathogens in children and domestic animals in a semirural community in Ecuador. Appl Environ Microbiol. 2016; 82(14): 4218–4224. doi: 10.1128/AEM.00795-16 PMID: 27208122.
10. Sarzosa M, Graham JP, Salinas L, Trueba G. Potential zoonotic transmission of *Giardia duodenalis* in semi-rural communities near Quito, Ecuador. Intern J Appl Res Vet Med. 2018; 16(1):1–6.
11. Cajamarca A, Criollo D, Solano R, Sacoto A, Mosquera L. Estudio experimental: prevención de parasitosis en escolares de una zona rural. Azuay, Ecuador. 2013– 2014. Rev Med HJCA. 2017; 9(2): 139–143. doi: 10.14410/2017.9.2.ao.23.
12. Helenbrook WD, Shields WM, Whipps CM. Characterization of *Blastocystis* species infection in humans and mantled howler monkeys, *Alouatta palliata aequatorialis*, living in close proximity to one another. Parasitol Res. 2015; 114(7): 2517–2525. doi: 10.1007/s00436-015-4451-x PMID: 25859926.
13. Calvopina M, Atherton R, Romero-Álvarez D, Castaneda B, Valverde-Muñoz G, Cevallos W, et al. Identification of intestinal parasite infections and associated risk factors in indigenous Tsáchilas communities of Ecuador. Int J Acad Med. 2019; 5(3): 171–179.
14. Lowenstein C, Vasco K, Sarzosa S, Salinas L, Torres A, Perry MJ, et al. Determinants of childhood zoonotic enteric infections in a semirural community of Quito, Ecuador. Am J Trop Med Hyg. 2020; 102(6): 1269–1278. doi: 10.4269/ajtmh.19-0690 PMID: 32228797.
15. Levecke B, Dreesen L, Barrionuevo-Samaniego M, Ortiz WB, Praet N, Brandt J, et al. Molecular differentiation of Entamoeba spp. in a rural community of Loja province, South Ecuador. Trans R Soc Trop Med Hyg. 2011; 105(12): 737–739. doi: 10.1016/j.trstmh.2011.08.010 PMID: 21981992.
16. Guevara Á, Vicuña Y, Costales D, Vivero S, Anselmi M, Bisoffi Z, et al. Use of real-time polymerase chain reaction to differentiate between pathogenic Entamoeba histolytica and the nonpathogenic Entamoeba dispar in Ecuador. Am J Trop Med Hyg. 2019; 100(1): 81–82. doi: 10.4269/ajtmh.17-1022 PMID: 30398142.
