# Supplementary material for: Prevalence and associated risk factors of intestinal parasites among schoolchildren in Ecuador, with emphasis on the molecular diversity of Giardia duodenalis, Blastocystis sp. and Enterocytozoon bieneusi
Source: PLoS Negl Trop Dis. 2023 May 24;17(5):e0011339. doi: 10.1371/journal.pntd.0011339 (PMC10243618; doi:10.1371/journal.pntd.0011339)
Supplement: S3 Table — (DOCX) [file pntd.0011339.s003.docx]

Table S3. English version of the standardized epidemiological questionnaire employed.

| **Variable** | **Category and State code** |
| --- | --- |
| School sample | String variable (free text) |
| Sampling kit ID | String variable (free text) |
| Sampling date | Date variable |
| Country of birth | Sampling date |
| Municipality of residence | Sampling date |
| Gender | Woman (1)  Men (2) |
| Age | Integer variable (number) |
| Number of siblings | Integer variable (number) |
| Number of relatives residing at home | Integer variable (number) |
| Consumption of unsafe water | Yes (1)  No (2) |
| Hand and fruit/vegetable washing | Yes (1)  No (2) |
| Contact with domestic animals | Yes (1)  No (2) |
| Playing outdoors | Yes (1)  No (2) |
| Diarrhoea | Yes (1)  No (2) |
| Constipation | Yes (1)  No (2) |
| Abdominal pain | Yes (1)  No (2) |
| Vomiting | Yes (1)  No (2) |
| Weight loss | Yes (1)  No (2) |
| Allergic manifestations | Yes (1)  No (2) |
| Teeth grinding | Yes (1)  No (2) |
| Anal itching | Yes (1)  No (2) |
| Abdominal distention | Yes (1)  No (2) |
| Flatulence | Yes (1)  No (2) |
