# Supplementary material for: Prevalence and associated risk factors of intestinal parasites among schoolchildren in Ecuador, with emphasis on the molecular diversity of Giardia duodenalis, Blastocystis sp. and Enterocytozoon bieneusi
Source: PLoS Negl Trop Dis. 2023 May 24;17(5):e0011339. doi: 10.1371/journal.pntd.0011339 (PMC10243618; doi:10.1371/journal.pntd.0011339)
Supplement: S4 Table — (DOCX) [file pntd.0011339.s004.docx]

**Table S4.** Oligonucleotides used for the molecular identification and/or characterization of the parasitic intestinal protist.

| **Target organism** | **Locus** | **Oligonucleotide** | **Sequence (5´–3´)** | **Reference** |
| --- | --- | --- | --- | --- |
| *Giardia duodenalis* | *ssu* rRNA | Probe | FAM–CCCGCGGCGGTCCCTGCTAG–BHQ1 | [1] |
|  |  | Gd-80F | GACGGCTCAGGACAACGGTT | [1] |
|  |  | Gd-127R | TTGCCAGCGGTGTCCG | [1] |
|  | *gdh* | GDHeF | TCAACGTYAAYCGYGGYTTCCGT | [2] |
|  |  | GDHiF | CAGTACACCTCYGCTCTCGG | [2] |
|  |  | GDHiR | GTTRTCCTTGCACATCTCC | [2] |
|  | *bg* | G7_F | AAGCCCGACGACCTCACCCGCAGTGC | [3] |
|  |  | G759_R | GAGGCCGCCCTGGATCTTCGAGACGAC | [3] |
|  |  | G99_F | GAACGAACGAGATCGAGGTCCG | [3] |
|  |  | G609_R | CTCGACGAGCTTCGTGTT | [3] |
|  | *tpi* | AL3543 | AAATIATGCCTGCTCGTCG | [4] |
|  |  | AL3546 | CAAACCTTITCCGCAAACC | [4] |
|  |  | AL3544 | CCCTTCATCGGIGGTAACTT | [4] |
|  |  | AL3545 | GTGGCCACCACICCCGTGCC | [4] |
| *Entamoeba histolytica* | *ssu* rRNA | Probe | FAM–TCATTGAATGAATTGGCCATTT–MGB | [5] |
| *Entamoeba dispar* |  | Probe | VIC–TTACTTACATAAATTGGCCACTTTG–MGB | [5] |
| *Entamoeba histolytica*/*dispar* |  | Ehd-239F | ATTGTCGTGGCATCCTAACTCA | [6] |
|  |  | Ehd-88R | GCGGACGGCTCATTATAACA | [6] |
| *Cryptosporidium* spp. | *ssu* rRNA | CR-P1 | CAGGGAGGTAGTGACAAGAA | [7] |
|  |  | CR-P2 | TCAGCCTTGCGACCATACTC | [7] |
|  |  | CR-P3 | ATTGGAGGGCAAGTCTGGTG | [7] |
|  |  | CPB-DIAGR | TAAGGTGCTGAAGGAGTAAGG | [7] |
| *Blastocystis* sp. | *ssu* rRNA | BhRDr | GAGCTTTTTAACTGCAACAACG | [8] |
|  |  | RD5 | ATCTGGTTGATCCTGCCAGT | [8] |
| *Enterocytozoon bieneusi* | ITS | EBITS3 | GGTCATAGGGATGAAGAG | [9] |
|  |  | EBITS4 | TTCGAGTTCTTTCGCGCTC | [9] |
|  |  | EBITS1 | GCTCTGAATATCTATGGCT | [9] |
|  |  | EBITS2.4 | ATCGCCGACGGATCCAAGTG | [9] |

*bg*: β-giardin (bg); *gdh*: Glutamate dehydrogenase; ITS: Internal Transcribed Spacer; *ssu* rRNA: Small subunit ribosomal RNA; *tpi*: Triose Phosphate Isomerase. ^1^Available in the Reference section of the main body of the manuscript.

**References**

1. Verweij JJ, Schinkel J, Laeijendecker D, van Rooyen MA, van Lieshout L, Polderman AM. Real-time PCR for the detection of *Giardia lamblia*. Mol Cell Probes. 2003; 17(5): 223–225. doi: 10.1016/s0890-8508(03)00057-4 PMID: 14580396.
2. Read CM, Monis PT, Thompson RC. Discrimination of all genotypes of *Giardia duodenalis* at the glutamate dehydrogenase locus using PCR-RFLP. Infect Genet Evol. 2004; 4(2): 125–130. doi: 10.1016/j.meegid.2004.02.001 PMID: 15157630.
3. Lalle M, Pozio E, Capelli G, Bruschi F, Crotti D, Cacciò SM. Genetic heterogeneity at the beta-giardin locus among human and animal isolates of *Giardia duodenalis* and identification of potentially zoonotic subgenotypes. Int J Parasitol. 2005; 35(2): 207-13. doi: 10.1016/j.ijpara.2004.10.022 PMID: 15710441.
4. Sulaiman IM, Fayer R, Bern C, Gilman RH, Trout JM, Schantz PM, et al. Triosephosphate isomerase gene characterization and potential zoonotic transmission of *Giardia duodenalis*. Emerg Infect Dis. 2003; 9(11): 1444–1452. doi: 10.3201/eid0911.030084 PMID: 14718089.
5. Gutiérrez-Cisneros MJ, Cogollos R, López-Vélez R, Martín-Rabadán P, Martínez-Ruiz R, Subirats M, et al. Application of real-time PCR for the differentiation of *Entamoeba histolytica* and *E. dispar* in cyst-positive faecal samples from 130 immigrants living in Spain. Ann Trop Med Parasitol. 2010; 104(2): 145–149. doi: 10.1179/136485910X12607012373759 PMID: 20406581.
6. Verweij JJ, Oostvogel F, Brienen EA, Nang-Beifubah A, Ziem J, Polderman AM. Prevalence of *Entamoeba histolytica* and *Entamoeba dispar* in northern Ghana. Trop Med Int Health. 2003; 8(12): 1153–1156. doi: 10.1046/j.1360-2276.2003.01145.x PMID: 14641852.
7. Tiangtip R, Jongwutiwes S. Molecular analysis of *Cryptosporidium* species isolated from HIV-infected patients in Thailand. Trop Med Int Health. 2002; 7(4): 357–364. doi: 10.1046/j.1365-3156.2002.00855.x PMID: 11952952.
8. Scicluna SM, Tawari B, Clark CG. DNA barcoding of *Blastocystis*. Protist. 2006; 157(1): 77–85. doi: 10.1016/j.protis.2005.12.001 PMID: 16431158.
9. Buckholt MA, Lee JH, Tzipori S. Prevalence of *Enterocytozoon bieneusi* in swine: an 18-month survey at a slaughterhouse in Massachusetts. Appl Environ Microbiol. 2002; 68(5): 2595–2599. doi: 10.1128/AEM.68.5.2595-2599.2002.
