# Supplementary material for: Prevalence and associated risk factors of intestinal parasites among schoolchildren in Ecuador, with emphasis on the molecular diversity of Giardia duodenalis, Blastocystis sp. and Enterocytozoon bieneusi
Source: PLoS Negl Trop Dis. 2023 May 24;17(5):e0011339. doi: 10.1371/journal.pntd.0011339 (PMC10243618; doi:10.1371/journal.pntd.0011339)
Supplement: S5 Table — (DOCX) [file pntd.0011339.s005.docx]

**Table S5.** PCR cycling conditions used for the molecular identification and/or characterization of the parasitic intestinal protist.

|  |  | **Temperature and time** | | | |  |  |  |
| --- | --- | --- | --- | --- | --- | --- | --- | --- |
| **Target organism** | **Locus** | **Initial denaturation** | **Denaturation** | **Annealing** | **Extension** | **No. cycles** | **Final extension** | **Reference** |
| *Giardia duodenalis* | *ssu* rRNA | 95°C 15 min | 95°C 15 s | 60°C 1 min | 72°C 30 s | 45 | – | [1] |
|  | *gdh* | 95°C 3 min | 95°C 30 s | 55°C 30 s | 72°C 1 min | 35 | 72°C 7 min | [2] |
|  | *bg* | 95°C 7 min | 95°C 30 s | 65/55°C 30 s | 72°C 1 min | 35 | 72°C 7 min | [3] |
|  | *tpi* | 94°C 5 min | 94°C 45 s | 50°C 45 s | 72°C 1 min | 35 | 72°C 10 min | [4] |
| *Entamoeba histolytica* | *ssu* rRNA | 95°C 15 min | 95°C 15 s | 60°C 1 min | 72°C 30 s | 45 | – | [5] |
| *Cryptosporidium* spp. | *ssu* rRNA | 94°C 3 min | 94°C 40 s | 50°C 40 s | 72°C 1 min | 35 | 72°C 10 min | [6] |
| *Blastocystis* sp. | *ssu* rRNA | 95°C 3 min | 94°C 1 min | 59°C 1 min | 72°C 1 min | 30 | 72°C 2 min | [7] |
| *Enterocytozoon bieneusi* | ITS | 94°C 3 min | 94°C 30 s | 55/57°C 30 s | 72°C 40 s | 35 | 72°C 10 min | [8] |

*bg*: β-giardin (bg); *gdh*: Glutamate dehydrogenase; ITS: Internal transcribed spacer; *gp60*: 60 kDa glycoprotein; *ssu* rRNA: Small subunit ribosomal RNA; *tpi*: Triose phosphate isomerase.

**References**

1. Verweij JJ, Schinkel J, Laeijendecker D, van Rooyen MA, van Lieshout L, Polderman AM. Real-time PCR for the detection of *Giardia lamblia*. Mol Cell Probes. 2003; 17(5): 223–225. doi: 10.1016/s0890-8508(03)00057-4 PMID: 14580396.
2. Lalle M, Pozio E, Capelli G, Bruschi F, Crotti D, Cacciò SM. Genetic heterogeneity at the beta-giardin locus among human and animal isolates of *Giardia duodenalis* and identification of potentially zoonotic subgenotypes. Int J Parasitol. 2005; 35(2): 207-13. doi: 10.1016/j.ijpara.2004.10.022 PMID: 15710441.
3. Sulaiman IM, Fayer R, Bern C, Gilman RH, Trout JM, Schantz PM, et al. Triosephosphate isomerase gene characterization and potential zoonotic transmission of *Giardia duodenalis*. Emerg Infect Dis. 2003; 9(11): 1444–1452. doi: 10.3201/eid0911.030084 PMID: 14718089.
4. Gutiérrez-Cisneros MJ, Cogollos R, López-Vélez R, Martín-Rabadán P, Martínez-Ruiz R, Subirats M, et al. Application of real-time PCR for the differentiation of *Entamoeba histolytica* and *E. dispar* in cyst-positive faecal samples from 130 immigrants living in Spain. Ann Trop Med Parasitol. 2010; 104(2): 145–149. doi: 10.1179/136485910X12607012373759 PMID: 20406581.
5. Verweij JJ, Oostvogel F, Brienen EA, Nang-Beifubah A, Ziem J, Polderman AM. Prevalence of *Entamoeba histolytica* and *Entamoeba dispar* in northern Ghana. Trop Med Int Health. 2003; 8(12): 1153–1156. doi: 10.1046/j.1360-2276.2003.01145.x PMID: 14641852.
6. Tiangtip R, Jongwutiwes S. Molecular analysis of *Cryptosporidium* species isolated from HIV-infected patients in Thailand. Trop Med Int Health. 2002; 7(4): 357–364. doi: 10.1046/j.1365-3156.2002.00855.x PMID: 11952952.
7. Scicluna SM, Tawari B, Clark CG. DNA barcoding of *Blastocystis*. Protist. 2006; 157(1): 77–85. doi: 10.1016/j.protis.2005.12.001 PMID: 16431158.
8. Buckholt MA, Lee JH, Tzipori S. Prevalence of *Enterocytozoon bieneusi* in swine: an 18-month survey at a slaughterhouse in Massachusetts. Appl Environ Microbiol. 2002; 68(5): 2595–2599. doi: 10.1128/AEM.68.5.2595-2599.2002.
