# Supplementary material for: Prevalence and associated risk factors of intestinal parasites among schoolchildren in Ecuador, with emphasis on the molecular diversity of Giardia duodenalis, Blastocystis sp. and Enterocytozoon bieneusi
Source: PLoS Negl Trop Dis. 2023 May 24;17(5):e0011339. doi: 10.1371/journal.pntd.0011339 (PMC10243618; doi:10.1371/journal.pntd.0011339)
Supplement: S6 Table — Adjusted Odds Ratios (AORs) and 95% Confidential Intervals (95% CI) are indicated. (DOCX) [file pntd.0011339.s006.docx]

**Table S6**. Multivariate logistic regression analysis for intestinal parasitism in the surveyed population according to the municipality of origin. Adjusted Odds Ratios (AORs) and 95% Confidential Intervals (95% CI) are indicated.

|  |  | **All** | | | | **GAE (*n* = 115)** | | | | **Penipe (*n* = 96)** | | | | **Pallatanga (*n* = 161)** | | | |
| --- | --- | --- | --- | --- | --- | --- | --- | --- | --- | --- | --- | --- | --- | --- | --- | --- | --- |
|  | **Category** | ***n*** | **AOR** | **95% CI** | ***P*-value** | ***n*** | **AOR** | **95% CI** | ***P*-value** | ***n*** | **AOR** | **95% CI** | ***P*-value** | ***n*** | **AOR** | **95% CI** | ***P*-value** |
| Gender | Female | 200 | 0.891 | 0.534–1.486 | 0.657 | 64 | 0.567 | 0.245–1.314 | 0.186 | 50 | 0.921 | 0.208–4.076 | 0.913 | 86 | 1451 | 0.592–3.557 | 0.416 |
|  | Male | 172 | – | – | – | 51 | – | – | – | 46 | – | – | – | 75 | – | – | – |
| Age group (years) | 3–4 | 55 | 0.681 | 0.324–1.431 | 0.311 | 23 | 0.738 | 0.214–2.550 | 0.631 | 16 | 0.297 | 0.032–2.753 | 0.286 | 16 | 0.444 | 0.122–1.623 | 0.220 |
|  | 5–8 | 174 | 1.241 | 0.697–2.209 | 0.464 | 63 | 1101 | 0.412–2.943 | 0.848 | 45 | 2115 | 0.459–9.734 | 0.336 | 66 | 1536 | 0.581–4.053 | 0.388 |
|  | 9–11 | 143 | – | – | – | 29 | – | – | – | 35 | – | – | – | 79 | – | – | – |
| Contact with animals | Yes | 290 | 1.125 | 0.611–2.071 | 0.706 | 87 | 1538 | 0.572–4.132 | 0.393 | 83 | 0.859 | 0.111–6.625 | 0.884 | 120 | 0.821 | 0.290–2.327 | 0.711 |
|  | No | 82 | – | – | – | 28 | – | – | – | 13 | – | – | – | 41 | – | – | – |
| Washing fresh produce | Yes | 254 | – | – | – | 98 | – | – | – | 58 | – | – | – | 98 | – | – | – |
|  | No | 118 | 0.921 | 0.482–1.761 | 0.804 | 17 | 0.721 | 0.193–2.696 | 0.627 | 38 | 0.499 | 0.074–3.371 | 0.476 | 63 | 0.936 | 0.352–2.492 | 0.895 |
| Handwashing before eating | Yes | 261 | – | – | – | 92 | – | – | – | 64 | – | – | – | 105 | – | – | – |
|  | No | 111 | 1.919 | 0.944–3.904 | 0.072 | 23 | 1665 | 0.511–5.420 | 0.398 | 32 | 7473 | 0.607–92.06 | 0.116 | 56 | 2179 | 0.646–7.351 | 0.209 |
| Handwashing after bathing | Yes | 292 | – | – | – | 97 | – | – | – | 78 | – | – | – | 117 | – | – | – |
|  | No | 80 | 0.568 | 0.276–1.170 | 0.125 | 18 | 0.428 | 0.116–1.583 | 0.203 | 18 | 0.54 | 0.005–0.541 | 0.013 | 44 | 0.684 | 0.208–2.247 | 0.531 |
| Safe water | Yes | 163 | – | – | – | 81 | – | – | – | 36 | – | – | – | 46 | – | – | – |
|  | No | 209 | 1.093 | 0.621–1.924 | 0.758 | 34 | 1279 | 0.491–3.330 | 0.614 | 60 | 0.127 | 0.22–0.743 | 0.022 | 115 | 1971 | 0.766–5.071 | 0.159 |
| Plays outdoors | Yes | 160 | 1.027 | 0.606–1.742 | 0.921 | 41 | 0.617 | 0.260–1.466 | 0.274 | 50 | 4212 | 0.832–21.33 | 0.082 | 69 | 1638 | 0.623–4.310 | 0.317 |
|  | No | 212 | – | – | – | 74 | – | – | – | 46 | – | – | – | 92 | – | – | – |
| No. of siblings | 0 | 64 | – | – | – | 24 | – | – | – | 10 | – | – | – | 30 | – | – | – |
|  | 1–2 | 205 | 1.099 | 0.567–2.127 | 0.78 | 80 | 0.734 | 0.257–2.094 | 0.563 | 38 | 0.336 | 0.024–4.811 | 0.422 | 87 | 1669 | 0.552–5.049 | 0.364 |
| No. of relatives at home | 1 | 103 | 1.788 | 0.669–4.779 | 0.247 | 11 | 0.304 | 0.036–2.255 | 0.273 | 48 | 3704 | 0.233–58.90 | 0.354 | 44 | 3034 | 0.583–15.795 | 0.187 |
|  | 2–4 | 280 | – | – | – | 101 | – | – | – | 56 | – | – | – | 126 | – | – | – |
|  | >5 | 92 | 1.393 | 0.615–3.158 | 0.427 | 14 | 22.634 | 1.778–288.1 | 0.016 | 40 | 0.360 | 0.073–1.779 | 0.210 | 38 | 0.587 | 0.151–2.286 | 0.443 |
